# Molecular Dynamics Modeling of Potential Osteoarthritic Biomarkers

**DOI:** 10.3390/life15101506

**Published:** 2025-09-24

**Authors:** Joshua Mallets, Celeste Hicks, Tarun Goswami

**Affiliations:** 1 Biomedical, Industrial, and Human Factors Engineering, Wright State University, 3640 Col. Glenn Hwy, Dayton, OH 45435, USA; mallets.5@wright.edu (J.M.);; 2 Orthopaedic Surgery, Sports Medicine, and Rehabilitation, Wright State University, 3640 Col. Glenn Hwy, Dayton, OH 45435, USA

**Keywords:** osteoarthritis, biomarkers, proteins, molecular dynamics, stabilization

## Abstract

Osteoarthritis (OA) is one of the most common forms of arthritis and is commonly characterized by the breakdown of the hyaline cartilage and synovial fluid in joints. The body naturally responds by releasing proteins with specific functions to combat the degradation of the joint. The objective of the research undertaken in this study was to simulate a selection of these proteins from previous work in the literature to gather data on their energies. This was accomplished using the molecular dynamics software NAMD and VMD, in which each protein was simulated for 5 ps in water at three different temperatures. The simulations showed that at body temperature, orosomucoid-1 and complement component 4B had energies that stabilized significantly faster than the other proteins simulated, and alpha-2-macroglobulin had energies that stabilized significantly slower than the others. These outliers were further investigated by simulating them for 1 ns to reveal their molecular dynamics. Based on the data collected, it was proposed that the proteins that had faster stabilization times would be more stable biomarkers overall. Despite any limitations of the research performed, the novel work performed here provides a foundation for future work that could give clinical insight into the diagnosis and prognosis of individuals experiencing symptoms associated with osteoarthritis.

## 1. Introduction

### 1.1. Osteoarthritis

Osteoarthritis (OA) is one of the most common forms of arthritis [1,2]. It is often characterized, pathophysiologically, by the wear and deterioration of the hyaline cartilage found at the articular end of bones in diarthrodial joints [1]. Hyaline cartilage is a firm, smooth tissue that provides lubrication, reduces friction, and distributes load [3]. As it breaks down, the articular end of the bone becomes exposed and can abrade directly with the other bone [4]. This direct bone-on-bone movement has little to no protection or lubrication, causing friction, wear, destruction of the connective tissue, changes in the bone structure, inflammation in the synovium, and pain in the patient [2,4,5]. Several definitions of OA exist in its epidemiology, but it is common to define it radiographically, for which the Kellgren-Lawrence grading scheme of 0 to 4 is used [2]. OA affects approximately twenty-five percent of people over the age of eighteen and is found in approximately ten percent of men and thirteen percent of women aged sixty and older [2,4]. It can be caused by various factors classified as either systemic or local [2,4]. Examples of systemic factors that may cause OA include age, genetics, and diet, while local factors include individual biomechanics, injury, and obesity [2]. While several of these factors can play a role in the onset of OA, knowledge of the exact mechanism in which OA is induced and progresses, as well as methods for early detection, is lacking [1,4].

### 1.2. Synovial Fluid

Synovial fluid is a viscous fluid found in diarthrodial joints [6]. It provides lubrication and cushioning between the bones as well as nutrients for the hyaline cartilage [6]. Traditionally, OA has been classified as a non-inflammatory arthropathy; however, recent studies have identified synovitis, or inflammation of the synovium, in OA through medical imaging techniques such as magnetic resonance imaging and ultrasonography [7,8,9]. The literature suggests that the biological products associated with cartilage degradation in OA initiate synovial inflammation, and as OA progresses and degradation products increase, a cycle ensues, enhancing synovitis [9,10]. Synovitis in OA is associated with pain, synovial fluid degradation, accelerated cartilage degradation, and oxidative stress, thus strongly indicating that synovitis plays a role in the pathogenesis of osteoarthritic joints [7,8,9,10].

On a molecular level, the degradation of synovial fluid enhanced by OA and synovitis can be characterized by a reduced volume and molecular weight of hyaluronan [8]. Healthy synovial fluid contains a larger concentration of long hyaluronan strands. Under low shear rates, these strands of hyaluronan can interact and entangle, giving the synovial fluid an increased viscosity [11]. Conversely, high shear rates disentangle hyaluronan strands and lower the viscosity [11]. These viscoelastic rheological properties have been linked to the lubricating effects of synovial fluids, which are diminished with OA [11,12]. The network of healthy hyaluronan strands also reduces the diffusion of macromolecules, such as proteins, via steric hindrance [12]. When hyaluronan is degraded, macromolecules can diffuse more easily and interact with other molecules in the synovial fluid [12]. Thus, both the change in rheological properties and diffusivity can affect the molecular dynamics of proteins found in the synovial fluid.

### 1.3. Biomarker Identification

A biomarker, as defined by the United States Food and Drug Administration (FDA) and the National Institutes of Health (NIH) in the Biomarkers, EndpointS, and other Tools (BEST) Resource glossary, is “a defined characteristic that is measured as an indicator of normal biological processes, pathogenic processes, or responses to an exposure or intervention, including therapeutic interventions” [13]. Research conducted by Gobezie et al. aimed to compare the protein profiles of healthy synovial fluid and synovial fluid affected by OA, and from this comparison, identified potential biomarkers of OA. In this research, a protein analysis of the synovial fluid from twenty-one participants with early OA, twenty-one participants with late OA, and twenty control participants was conducted [1]. It was found that all samples shared a total of one hundred and thirty-five high-abundance proteins [1]. However, it was also found that sixteen proteins were upregulated specifically in OA [1]. In this test, the Wilcoxon rank sum test was used with an arbitrary *p*-value made small to limit the number of proteins. The upregulation of these proteins in people experiencing OA is supported by others as well [14,15,16,17,18,19,20,21,22,23,24]. Thus, due to their upregulation, these proteins meet the definition of and have been proposed to be biomarkers for early OA identification. The biomarkers identified by Gobezie et al. are shown in Table 1 along with the specificity, sensitivity, and *p*-values used to identify them [1]. 

Proteins have a wide ability to interact with other molecules that can influence their structure and function [25]. When the protein interacts with another molecule, whether it be water or any other molecule in the body, it forms bonds. The creation and destruction of these bonds with other molecules changes the energy of the protein [25]. The stability of a protein’s energy is critical as instability can lead to changes in the protein’s structure, function, or even the overall denaturation of the protein [25].

### 1.4. Scope

The proteins identified by Gobezie et al. in early OA synovial fluid are proposed to be useful biomarkers of OA but need more research before they can be used clinically. Currently, OA is primarily diagnosed radiographically; however, this diagnosis often comes later in disease progression. There is a need for a method of diagnosis that can be used earlier in OA symptom progression. Fluid biomarkers have been proposed as a minimally invasive method of diagnosing OA in early stages, which may improve treatment capabilities and disease prognosis. However, OA becomes increasingly volatile as the condition progresses, hyaline cartilage degrades, hyaluronan degrades, and associated conditions such as synovitis develop. Each of these factors can affect the presence, function, and stability of the identified proteins. Thus, to further the understanding of these biomarkers towards their use clinically, it is essential to investigate the molecular dynamics and quantify the overall stability of each protein. Additionally, comparing the molecular dynamics of multiple promising biomarkers may provide insight into the potentials of these biomarkers relative to each other, and could help guide future research into the most relevant biomarkers for clinical use.

This article aims to provide a novel and introductory look into the molecular dynamics of these biomarkers via an in-house molecular dynamics simulation protocol. This protocol includes simulating each biomarker in water at physiological temperature and comparing the time it takes for each of the protein’s energies to stabilize among one another. This protocol was expanded upon by simulating the proteins at two lower temperatures to observe the force field parameters of the program. This gives validity to the function of the program, ensuring that the simulations accurately model the molecular dynamics of the proteins studied in this article. One-nanosecond simulations were also performed on the three most relevant proteins to further investigate their molecular dynamics. From these simulations, valuable insight into the stability of each biomarker is presented, and its relationship to hypothetical clinical insight is suggested. This article is an introductory work, and as such, limitations are addressed.

## 2. Biomarkers

To simulate the identified proteins in Table 1, a viable model file must be available on the RCSB website [26,27,28,29,30,31,32,33,34]. However, some of the proteins either did not have a file or did not have a file that successfully ran in the simulation software. Thus, only nine of the biomarkers from Table 1 were able to be simulated. These nine proteins include albumin, alpha-1-microglobulin, alpha-2-macroglobulin, apolipoprotein E, complement component 3, haptoglobin, orosomucoid 1, complement component 4B, and retinol-binding protein 4, the functions of which are reviewed below.

Albumin binds to several ions such as Ca^2+^, Na^+^, K^+^, Zn^2+^, and Mg^2+^. It plays a role as a major transporter of zinc, calcium, and magnesium, and has an affinity for those ions from greatest to lowest in the same order, respectively. Albumin also binds to fatty acids, hormones, bilirubin, and drugs. However, the main role of albumin is the regulation of colloid osmotic pressure in the blood [35]. Additionally, albumin, when in the presence of globulin and hyaluronic acid, increases the lubrication abilities of synovial fluid in comparison to models in which there is no albumin [36]. In OA, the concentration of Na^+^ and Ca^2+^ increases while hyaluronic acid degrades [37]. This leads to less binding between albumin and hyaluronic acid and results in a less stable matrix, decreased lubrication, and an altered ratio between the albumin and hyaluronic acid [37].

Alpha-1-microglobulin is an antioxidant and has a role in tissue repair. In the blood, the antioxidant properties of alpha-1-microglobulin help to regulate red blood cell homeostasis and prevent damage. It also prevents the oxidation of low-density lipoprotein particles during inflammation. Outside of the blood, alpha-1-microglobulin prevents the oxidation of the extracellular matrix and helps reduce oxidized collagen [38]. One study found that alpha-1-microglobulin plays a role in antioxidative measures in bleeding-induced oxidative stress of knee synovial fluid [9]. While the study did not find a significant concentration increase in the protein in people with OA as compared to other oxidative knee conditions, it does identify that other studies have found an increase in the protein levels. It also recognizes that this discrepancy could be due to factors such as a different method used to isolate the protein and a different set of participants [15]. However, as stated, there are studies that do identify the elevation of alpha-1-microglobulin in OA synovial fluid [1,21].

Alpha-2-macroglobulin is able to inhibit the four classes of proteinases. The protein has a region that proteinases target. When the proteinase cleaves at this site, alpha-2-macroglobulin undergoes a conformational change, thus rendering the proteinase confined [39]. Literature suggests that this function of alpha-2-macroglobulin is employed in OA via the reduction in cartilage catabolic enzymes [16]. A signifying trait of OA is the degradation of cartilage at the articular end of bones. Cartilage catabolic enzymes break down the cartilage. Thus, elevated alpha-2-macroglobulin is suggested to negatively regulate these catabolic enzymes [16].

Apolipoprotein E is a protein that transports lipids mediated by lipoproteins. It is a key component in lipoprotein production and clearance and has amphipathic properties. Apolipoprotein E can also bind to several receptors, such as low-density lipoprotein receptors and immune receptors. Due to its role in lipid transport via lipoproteins, apolipoprotein E helps regulate lipid homeostasis in both tissues and plasma [40]. Additionally, it has been found that apolipoprotein E has inflammation regulation and tissue repair properties in synovial fluid. In contrast to other variants of apolipoprotein, it has been suggested that apolipoprotein E is synthesized locally in the synovial fluid, supporting the idea that apolipoprotein E has a role in immune response to antigens in the synovial fluid [41].

Complement component 3 is essential for the activation of the complement pathways. Once this pathway is activated, variations in complement component 3 can have various functions. For example, complement component 3B can bind to immune aggregates, and complement component 3a anaphylatoxin can mediate local inflammation. It also causes smooth muscle to contract, the permeability of blood vessels to increase, and histamines to be released [42]. The complement system is an integral part of the immune system, having a role in aspects such as phagocytosis, inflammation, and viral infections. As stated, complement component 3 has been found to activate the complement system, thus initiating the processes of the complement system [43]. Literature has also found that complement component 3 is produced locally in the synovial fluid and is connected to pain and other symptoms of OA [19].

Haptoglobin plays a role as an antioxidant and an antibacterial protein. It also has the function of capturing heme iron. This haptoglobin-heme complex is then excreted from the body via urine, thus protecting the kidney from damage that could be done by free heme iron [44]. In addition, the literature suggests that haptoglobin forms a complex with hyaluronic acid in synovial fluid. This complex, due to haptoglobin’s antioxidative properties, protects the matrix from oxidation. It is also stated that the protein’s presence in the synovial fluid is a response to injury or infection [45].

Orosomucoid 1 is a transport protein that helps regulate the immune system in acute-phase reactions [46]. It serves as one of the major acute-phase proteins, and thus its concentration is increased in injury or inflammation. Its transport function is in lipophilic and acidic drugs [47].

Complement component 4B has the function of binding to both immunoglobulins and immune complexes. It also helps increase the solubility of immune aggregates [48]. As stated before, the complement system is a key part of the immune system, having a role in phagocytosis, inflammation, and viral infections [43]. Complement component 4, from which complement component 4B is derived, is a part of both the classical and lectin pathways. Its role in the complement pathway is that of microbe destruction [49]. Individuals lacking complement component 4 are more likely to develop infections and autoimmune disorders [49].

Retinol-binding protein 4, as the name suggests, regulates the transport of retinol from the liver to peripheral tissue via blood plasma [50]. It is a part of the lipocalin family and transports vitamin A in addition to retinol. Literature has also identified retinol-binding protein 4 as an adipokine and thus has a role in metabolic disorders. This suggests that RBP4 has a role in OA, as obesity is a contributing factor in OA. Studies have found an increased level of RBP4 released by OA cartilage in the synovial fluid, therefore also suggesting that RBP4 is produced locally in the joints of those affected by OA [23].

Table 2 lists the nine biomarkers simulated. Each biomarker’s associated gene, weight in Daltons, and amino acid length are shown alongside their biomarker in Table 2.

## 3. Methods

The methodology for this article is largely based on an in-house protocol developed by Hicks et al. [60]. This protocol outlines the molecular dynamics simulation of proteins in water [60]. It uses the time taken for each energy to stabilize as a means of quantifying protein stability [60]. These stabilization times were then compared among the other proteins simulated in the study [60]. The protocol fits the aim of this study as the same means of protein comparison and stabilization assessment are desired.

The software used to simulate each protein was VMD, version 2.14 Win64, and NAMD, version 1.9.3 Windows OpenGL (32-bit Intel x86) (University of Illinois, IL USA). NAMD (Nanoscale Molecular Dynamics) is a software capable of performing molecular dynamics simulations of protein systems. VMD (Visual Molecular Dynamics) is a software used to visualize and analyze the results from molecular dynamics simulations. To load a protein into the NAMD simulation software, a .pdb file, downloaded from the RCSB databank, was required for each individual protein [26,27,28,29,30,31,32,33,34]. These proteins were then simulated for 5 ps in a water box at 310 K. The water boxes used were made of pure water and fitted to the dimensions of each protein, with an extra five to six Angstroms beyond the protein in each dimension. Data gathered from each protein was loaded into MATLAB, version R2022b, to plot the bond, electrostatic, kinetic, potential, and total energies over the 5 ps simulation. MATLAB was also used to create a box plot that compares the times that the proteins reached equilibrium for each of the different energies. Equilibrium time was calculated as the time it took for the energy to reach 98% or 99% of the magnitude of the average final energy.

The protocol was expanded upon by simulating the same proteins for 5 ps in a water box at 150 K and for 5 ps in a water box at 0 K. Equilibrium times were compared across the different temperatures for each biomarker’s energies. Each protein was simulated at 0 K and 150 K to observe and establish a baseline for the CHARMM36 force field parameters used in the program. The version of the open source CHARMM36 force field parameters used is titled “CHARMM36 All-Hydrogen Parameter File for Proteins” and was published in 2012 [61]. These parameters are important as they influence a protein’s conformation and stability at different temperatures, and the data obtained from the simulations at lower temperatures verify these parameters. The biomarkers with outlier energy times at body temperature (310 K) were simulated once more for 1 ns in a water box at 310 K. The noise in the energy data for these 1 ns simulations was analyzed and compared across biomarkers by calculating the root mean square (RMS), standard deviation, and relative standard deviation to RMS (percent change) values. These time intervals of 1 ns and 5 ps were all that was necessary to observe full stabilization of the energies. These time intervals were also considered to be the best available compromise between collected data size and simulation processing time due to computer hardware constraints.

## 4. Results

**Figure 1:**
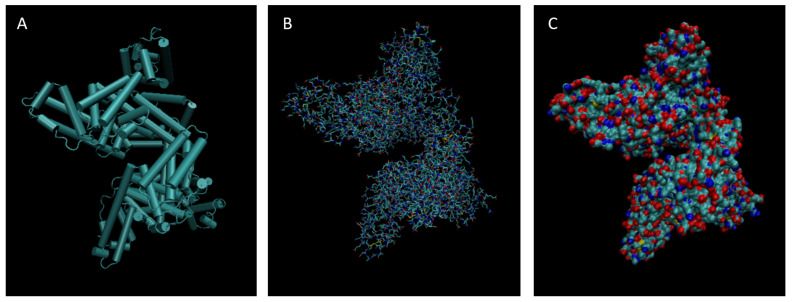
Cartoon (**A**), Bond (**B**), and Surface Model (**C**) of Albumin.

**Figure 2:**
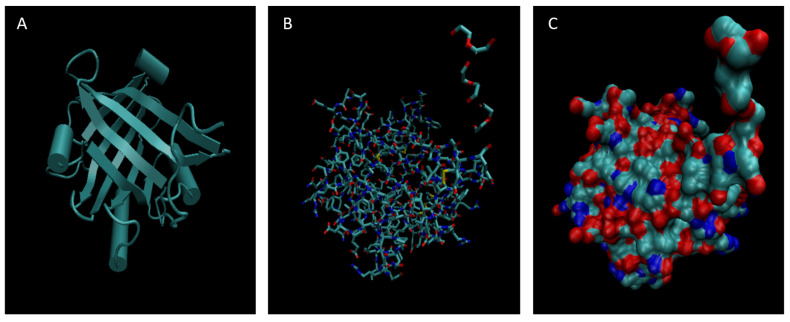
Cartoon (**A**), Bond (**B**), and Surface Model (**C**) of Alpha-1-microglobulin.

**Figure 3:**
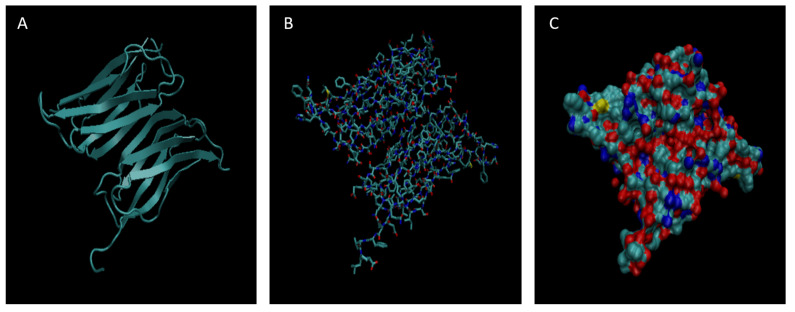
Cartoon (**A**), Bond (**B**), and Surface Model (**C**) of Alpha-2-macroglobulin.

**Figure 4:**
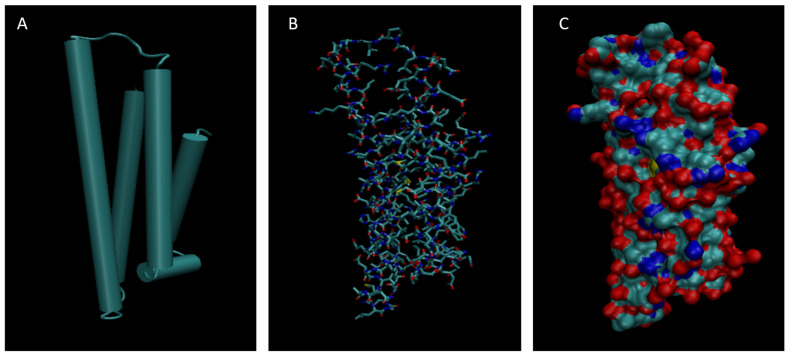
Cartoon (**A**), Bond (**B**), and Surface Model (**C**) of Apolipoprotein E.

**Figure 5:**
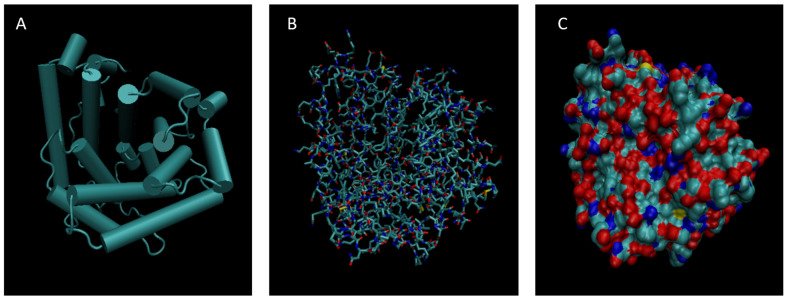
Cartoon (**A**), Bond (**B**), and Surface Model (**C**) of Complement Component 3.

**Figure 6:**
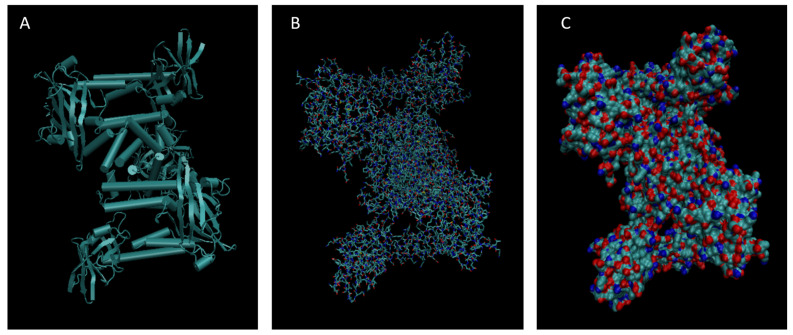
Cartoon (**A**), Bond (**B**), and Surface Model (**C**) of Haptoglobin.

**Figure 7:**
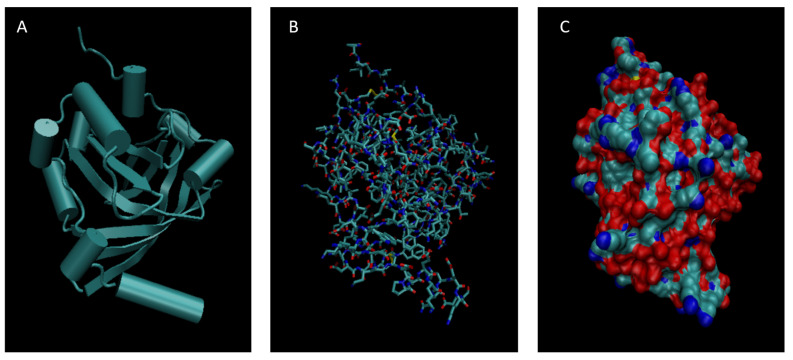
Cartoon (**A**), Bond (**B**), and Surface Model (**C**) of Orosomucoid 1.

**Figure 8:**
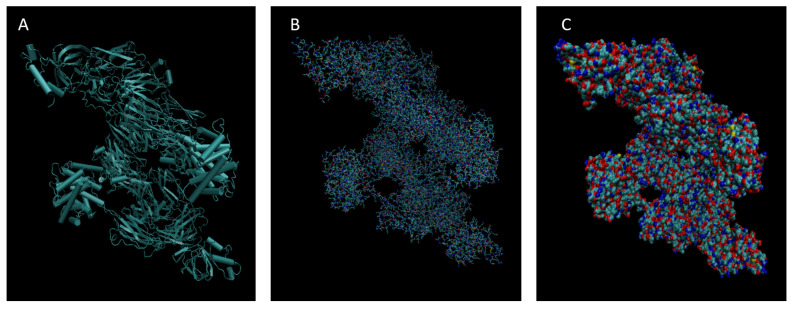
Cartoon (**A**), Bond (**B**), and Surface Model (**C**) of Complement Component 4B.

**Figure 9:**
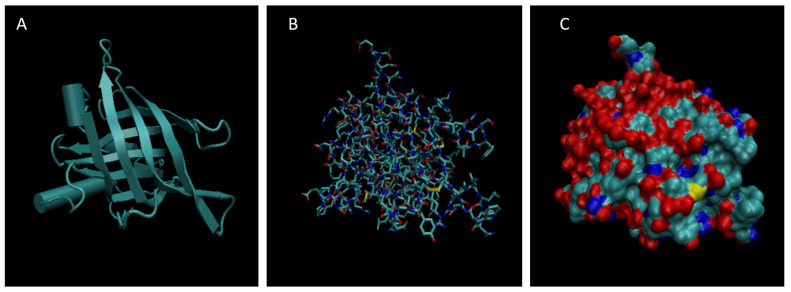
Cartoon (**A**), Bond (**B**), and Surface Model (**C**) of Retinol-binding protein 4.

Figure 1, Figure 2, Figure 3, Figure 4, Figure 5, Figure 6, Figure 7, Figure 8 and Figure 9 show a cartoon, bond, and surface model for each of the simulated proteins. The colors in both the bond and surface model represent the type of atom present, with white representing hydrogen, red oxygen, blue nitrogen, cyan carbon, and yellow sulfur. The cylinders in the cartoon model represent helices, and the solid ribbons represent beta sheets.

### 4.1. Biomarker Energies at 310 K

The bond, electrostatic, kinetic, total, and potential energies of each biomarker at 310 K over the duration of the 5 ps simulation were plotted in Figure 10, Figure 11, Figure 12, Figure 13, Figure 14, Figure 15, Figure 16, Figure 17 and Figure 18 below. The time it took each energy to reach equilibrium was also determined and compared across biomarkers, as shown by the boxplot in Figure 19.

Each energy was considered to be at equilibrium once it reached 98% or 99% of its final average magnitude (depending on the magnitude of the final energy reached). These equilibrium times for the biomarkers at 310 K are shown in Table 3 and compared by the box plot in Figure 19.

### 4.2. Biomarker Energies at 150 K

The effect of decreasing temperature on the stability of each biomarker was also investigated. The bond, electrostatic, kinetic, total, and potential energies of each biomarker at 150 K were plotted in Figure 20, Figure 21, Figure 22, Figure 23, Figure 24, Figure 25, Figure 26, Figure 27 and Figure 28 below. The time it took each energy to reach equilibrium was also determined and compared across biomarkers, as shown by the boxplot in Figure 29.

Each energy was considered to be at equilibrium once it reached 98% or 99% of its final average magnitude (depending on the magnitude of the final energy reached). These equilibrium times for the biomarkers at 150 K are shown in Table 4 and compared by the box plot in Figure 29.

### 4.3. Biomarker Energies at 0 K

The effect of decreasing temperature on protein stability was further investigated by simulating each biomarker at 0 K. The bond, electrostatic, kinetic, total, and potential energies of each biomarker at 0 K were plotted in Figure 30, Figure 31, Figure 32, Figure 33, Figure 34, Figure 35, Figure 36, Figure 37 and Figure 38 below. The time it took each energy to reach equilibrium was also determined and compared across biomarkers, as shown by the boxplot in Figure 39.

Each energy was considered to be at equilibrium once it reached 98% or 99% of its final average magnitude (depending on the magnitude of the final energy reached). These equilibrium times for the biomarkers at 0 K are shown in Table 5 and compared by the box plot in Figure 39.

### 4.4. Alpha-2 Macroglobulin, Orosomucoid-1, and Complement Component 4B 1ns Simulations

The kinetics of the three biomarkers with outlier energy times at body temperature (alpha-2-macroglobulin, orosomucoid-1, and complement component 4B) were analyzed by finding the root mean square (RMS), standard deviation, and relative standard deviation to RMS values for each energy in a 1 ns simulation. The energy plots obtained for each of these biomarkers from the 1 ns simulation are shown in Figure 40, Figure 41 and Figure 42.

Table 6, Table 7 and Table 8 show the obtained RMS, standard deviation, and relative standard deviation to RMS (percent change) for each biomarker’s energies.

## 5. Discussion

Thermodynamic stability is an important characteristic of biomarkers, as changes in thermodynamics can result in conformational changes, functional changes, and denaturation of the protein entirely [25]. Therefore, quantifying the thermodynamic energy and using it to characterize each protein relative to one another is insightful in the context of OA. Analyzing data from the simulations, it was observed that all the proteins reached their thermodynamic equilibrium within their 5 ps simulation. However, the box plot for the physiologically relevant 310 K simulation shows that the electrostatic energy of complement component 4B, as well as the potential energy of orosomucoid-1, stabilized faster than the other biomarkers. On the contrary, the plot shows that both the potential and total energy of alpha-2-macroglobulin took longer to reach equilibrium than the rest of the proteins. It is proposed that proteins that stabilize faster would be more stable proteins in comparison to those that stabilize slower [60]. Thus, orosomucoid-1 and complement component 4B would be more stable and alpha-2-macroglobulin less stable than the other proteins simulated.

### 5.1. Lower Temperature Simulations

The stability of each biomarker was further studied by investigating the reactions of each biomarker’s energies to changing temperatures. Biomarkers with outlier energy equilibrium times at lower temperatures, as determined by box plot analysis, included retinol-binding protein 4 and apolipoprotein E at 150 K and alpha-1-microglobulin and complement component 4B at 0 K. At 150 K and 0 K, alpha-2-macroglobulin and orosomucoid-1 no longer had any energy outliers. This change suggests that alpha-2-macroglobulin and orosomucoid-1 may have had conformation changes induced by the reduced temperatures, which led to changes in their stabilities. For alpha-2-macroglobulin, this change resulted in increased stability, while for orosomucoid-1, this change led to decreased stability. Complement component 4B’s electrostatic energy was significantly more stable than that of the other biomarkers at 310 K, but was of average stability at 150 K and 0 K. However, complement component 4B’s potential and total energies had high stability at 0 K and average stability at 310 K and 150 K. These changes in the stabilities of different energies suggest specific temperature-related conformational changes in complement component 4B, which may make it more stable near 310 K and 0 K, but less stable at some temperatures between these. Alpha-1-microglobulin had decreased stability at 0 K, and retinol-binding protein 4 and apolipoprotein E had decreased stability at 150 K, similarly suggesting stability-altering conformational changes in these proteins as well. The remaining proteins (albumin, haptoglobin, and complement component 3) had average stability at each temperature, suggesting that any temperature-induced conformational changes these proteins experienced were not significantly more than expected.

**Figure 10:**
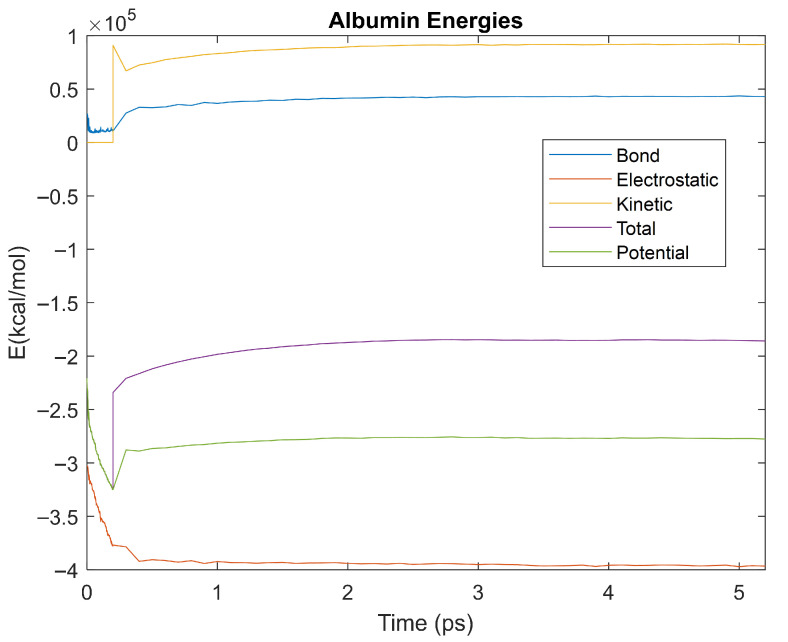
A plot of Bond, Electrostatic, Kinetic, Total, and Potential Energies for Albumin over the 310 K 5 ps simulation.

**Figure 11:**
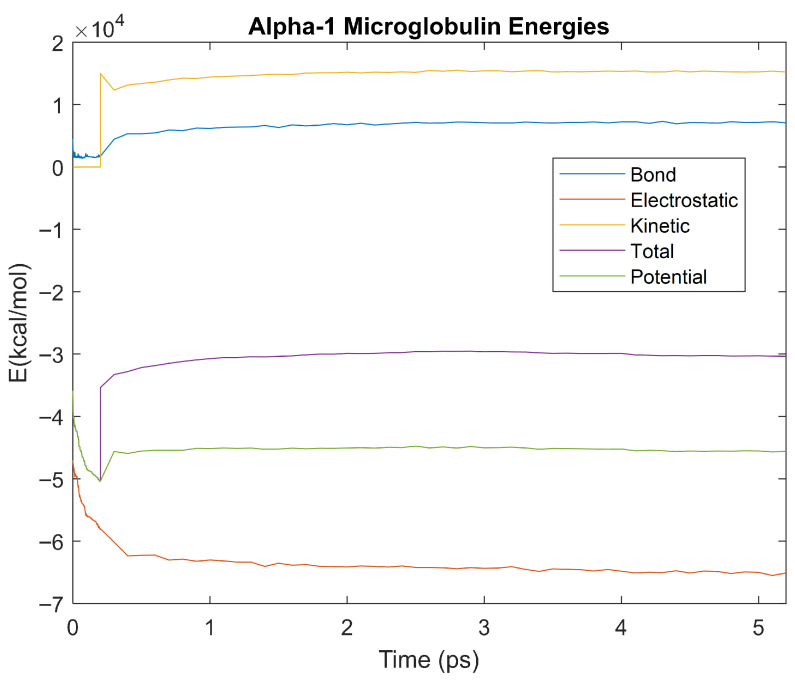
A plot of Bond, Electrostatic, Kinetic, Total, and Potential Energies for Alpha-1 Microglobulin over the 310 K 5 ps simulation.

**Figure 12:**
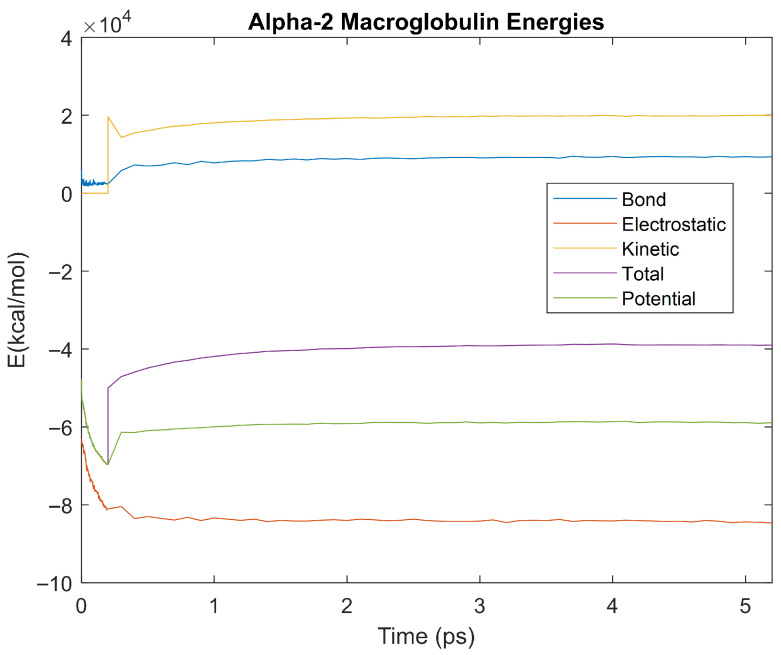
A plot of Bond, Electrostatic, Kinetic, Total, and Potential Energies for Alpha-2 Macroglobulin over the 310 K 5 ps simulation.

**Figure 13:**
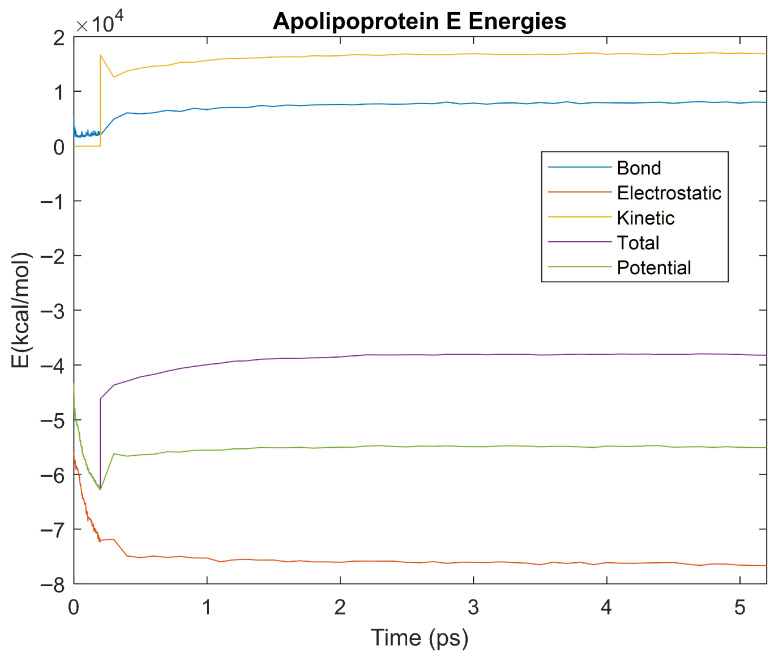
A plot of Bond, Electrostatic, Kinetic, Total, and Potential Energies for Apolipoprotein E over the 310 K 5 ps simulation.

**Figure 14:**
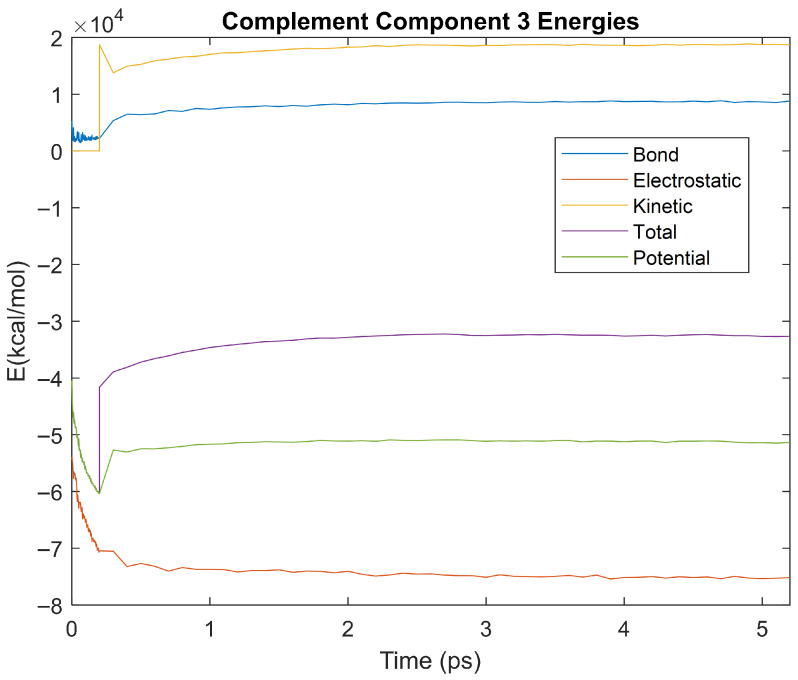
A plot of Bond, Electrostatic, Kinetic, Total, and Potential Energies for Complement Component 3 over the 310 K 5 ps simulation.

**Figure 15:**
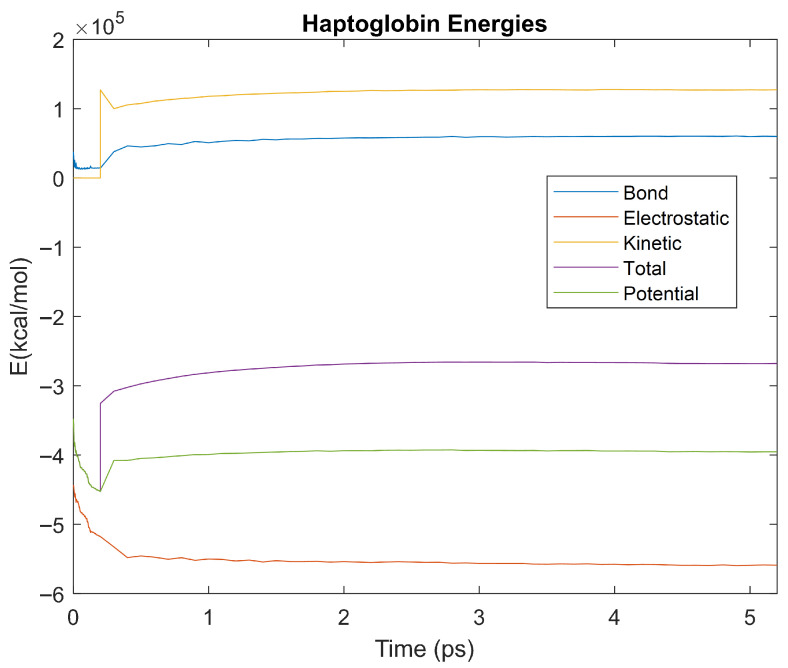
A plot of Bond, Electrostatic, Kinetic, Total, and Potential Energies for Haptoglobin over the 310 K 5 ps simulation.

**Figure 16:**
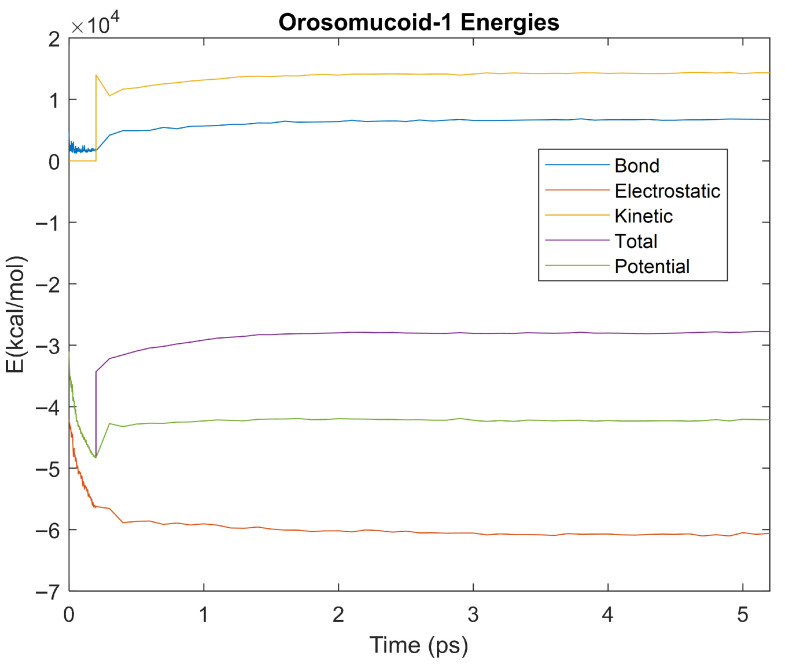
A plot of Bond, Electrostatic, Kinetic, Total, and Potential Energies for Orosomucoid 1 over the 310 K 5 ps simulation.

**Figure 17:**
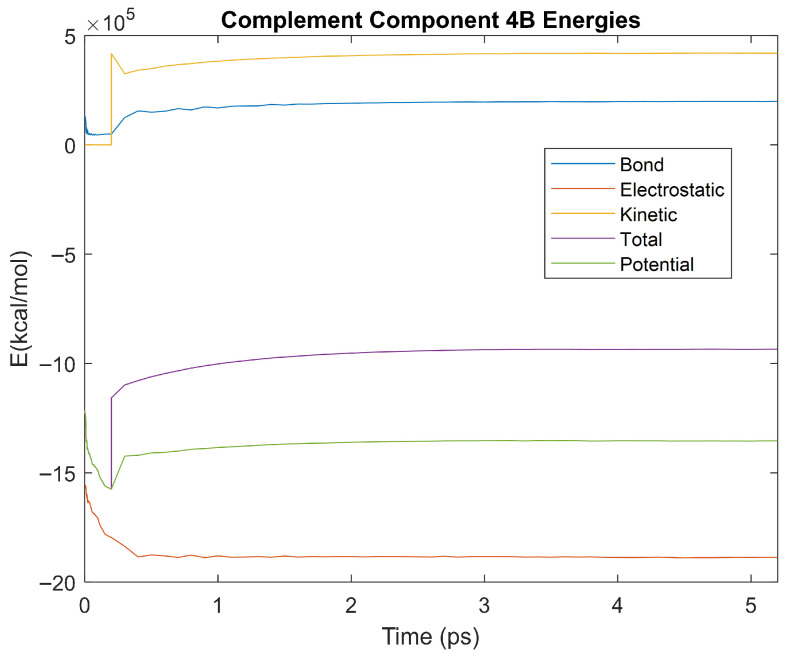
A plot of Bond, Electrostatic, Kinetic, Total, and Potential Energies for Complement Component 4B over the 310 K 5 ps simulation.

**Figure 18:**
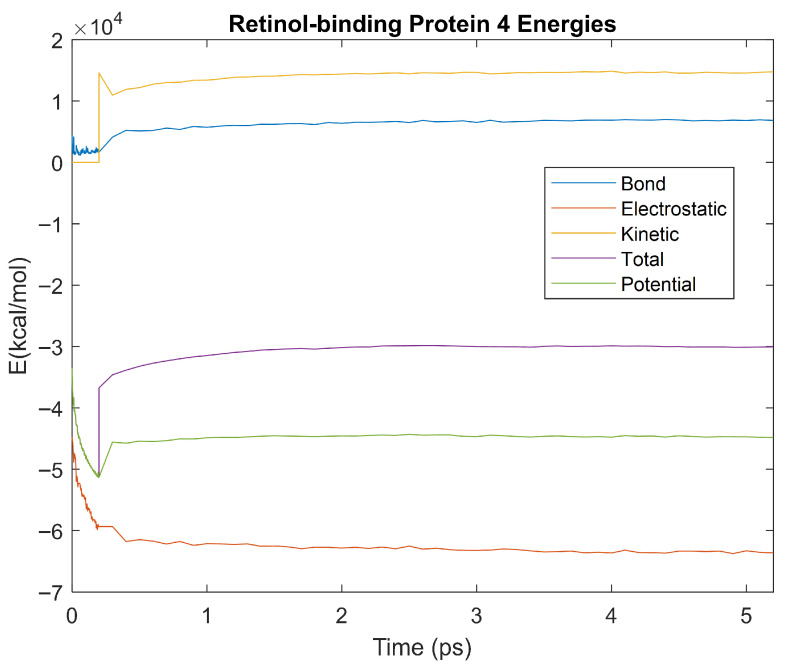
A plot of Bond, Electrostatic, Kinetic, Total, and Potential Energies for Retinol-binding Protein 4 over the 310 K 5 ps simulation.

**Figure 19:**
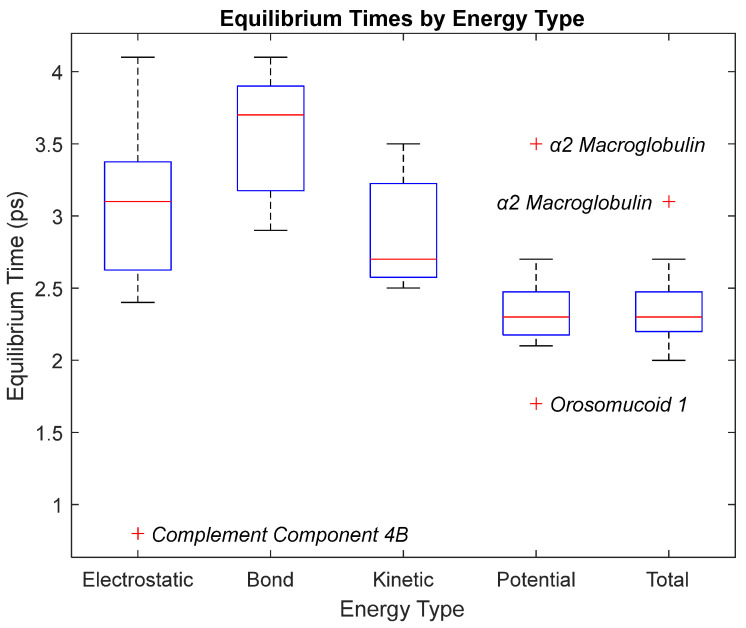
A box plot of the equilibrium times for each energy type. Data for this plot can be seen in Table 3. The red line represents the median time, the blue box is the interquartile range (25th–75th percentile), and the ends of the whiskers represent the 0th and 100th percentile, also called the maximum and minimum. Outliers are marked with red crosses and are labeled with their respective biomarker.

**Figure 20:**
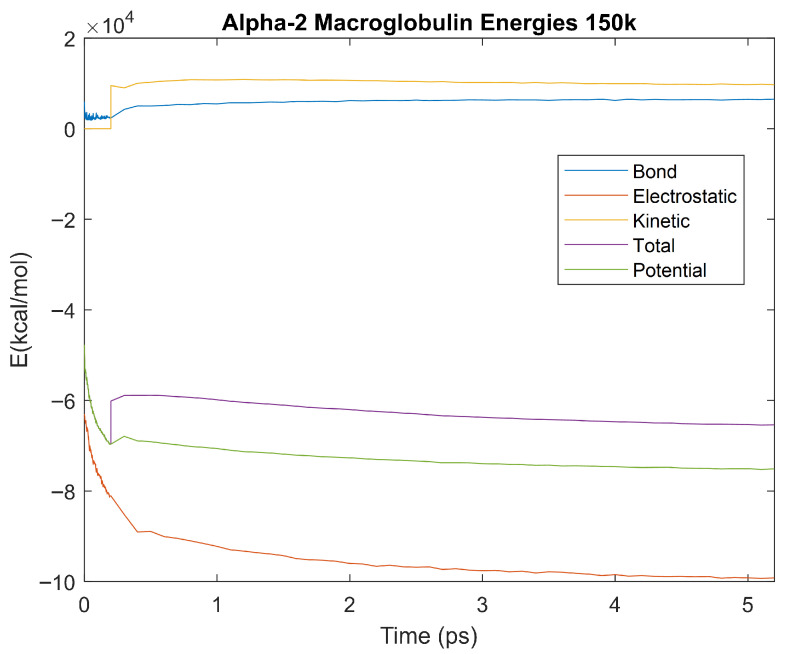
A plot of Bond, Electrostatic, Kinetic, Total, and Potential Energies for Alpha-2 Macroglobulin over the 150 K 5 ps simulation.

**Figure 21:**
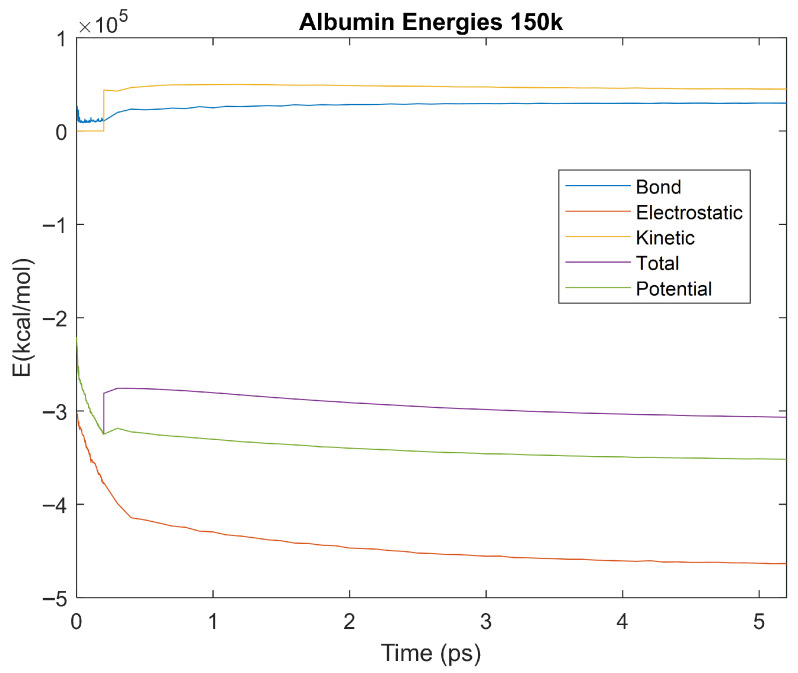
A plot of Bond, Electrostatic, Kinetic, Total, and Potential Energies for Albumin over the 150 K 5 ps simulation.

**Figure 22:**
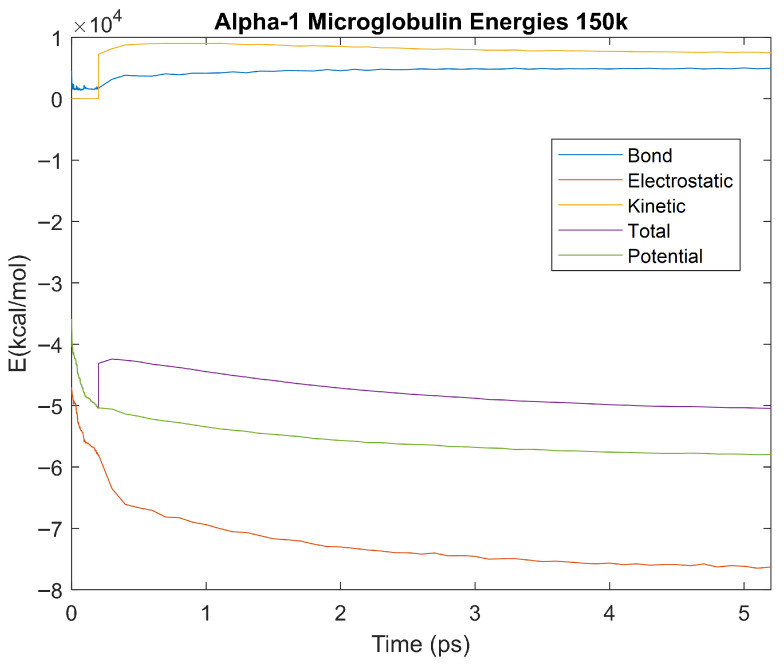
A plot of Bond, Electrostatic, Kinetic, Total, and Potential Energies for Alpha-1 Microglobulin over the 150 K 5 ps simulation.

**Figure 23:**
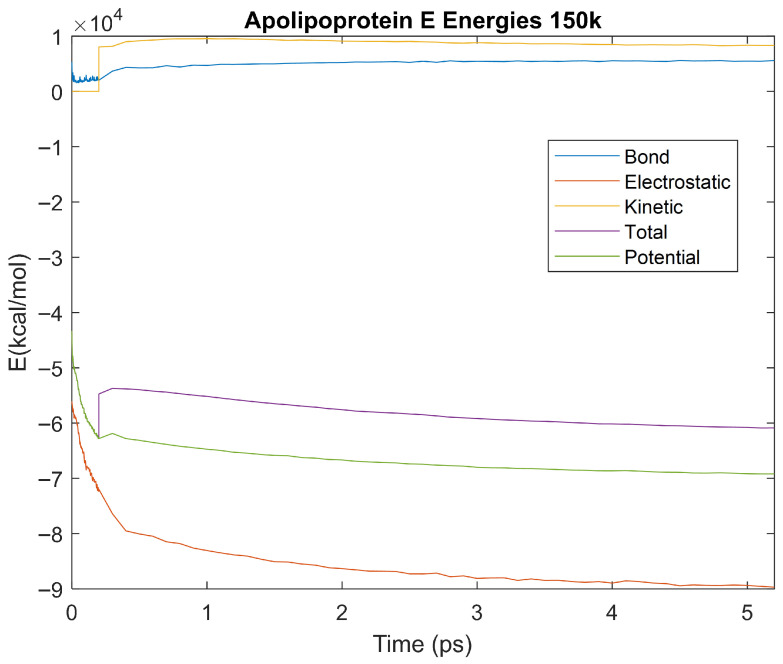
A plot of Bond, Electrostatic, Kinetic, Total, and Potential Energies for Apolipoprotein E over the 150 K 5 ps simulation.

**Figure 24:**
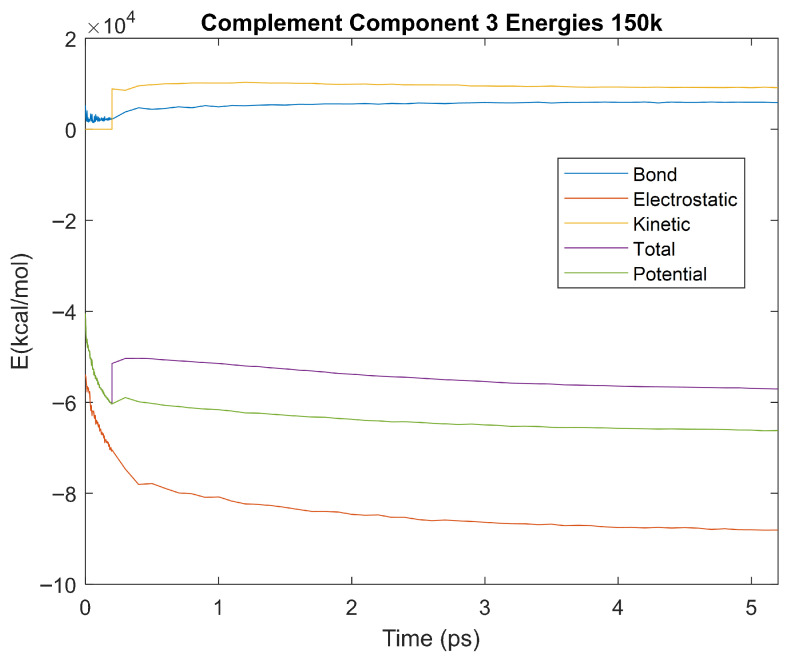
A plot of Bond, Electrostatic, Kinetic, Total, and Potential Energies for Complement Component 3 over the 150 K 5 ps simulation.

**Figure 25:**
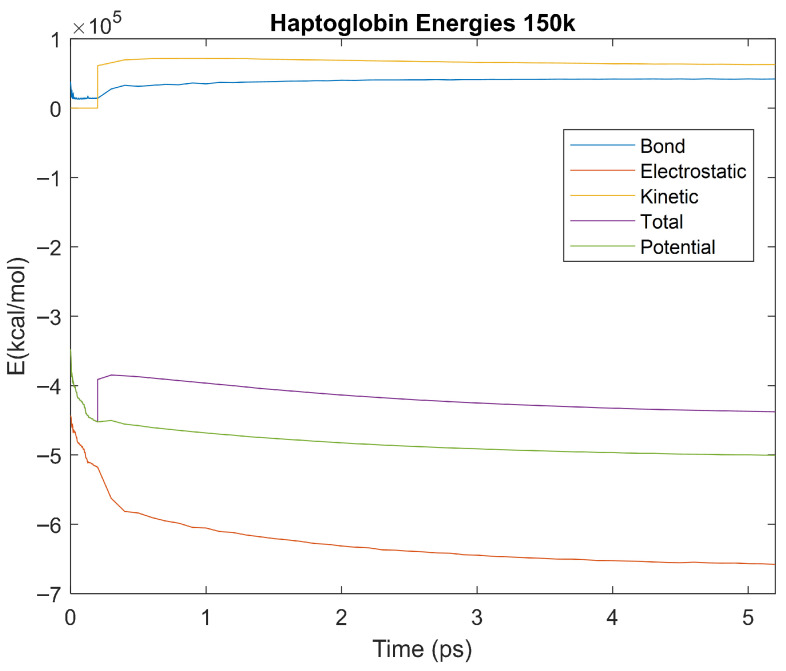
A plot of Bond, Electrostatic, Kinetic, Total, and Potential Energies for Haptoglobin over the 150 K 5 ps simulation.

**Figure 26:**
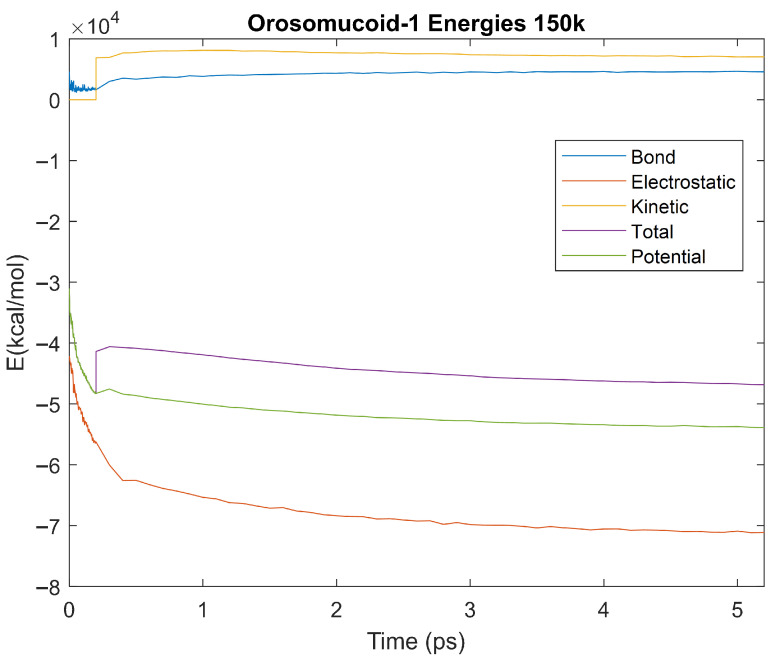
A plot of Bond, Electrostatic, Kinetic, Total, and Potential Energies for Orosomucoid-1 over the 150 K 5 ps simulation.

**Figure 27:**
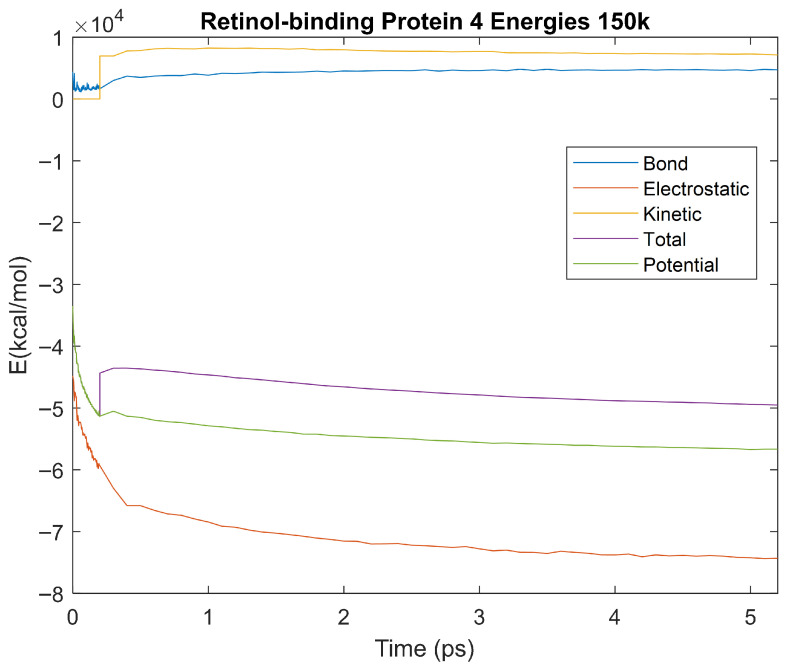
A plot of Bond, Electrostatic, Kinetic, Total, and Potential Energies for Retinol-Binding Protein 4 over the 150 K 5 ps simulation.

**Figure 28:**
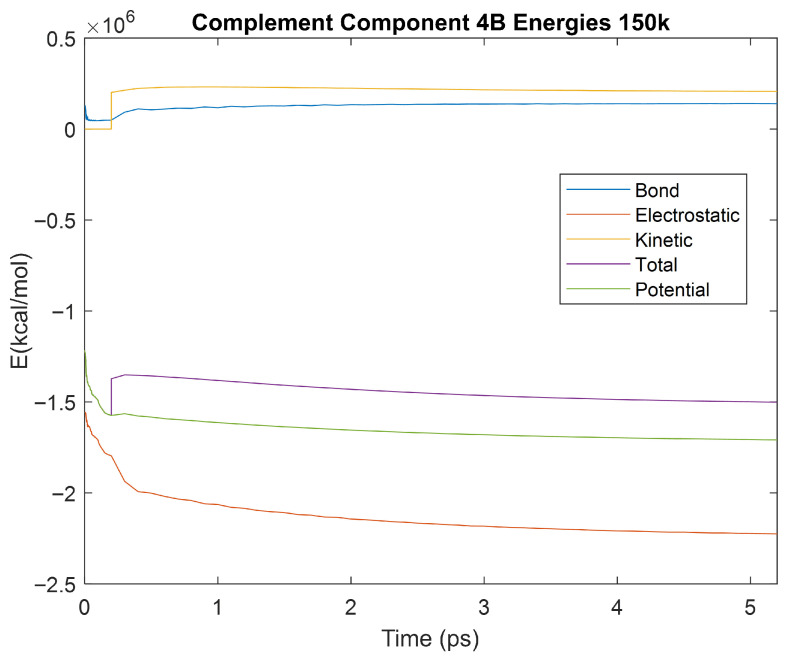
A plot of Bond, Electrostatic, Kinetic, Total, and Potential Energies for Complement Component 4B over the 150 K 5 ps simulation.

**Figure 29:**
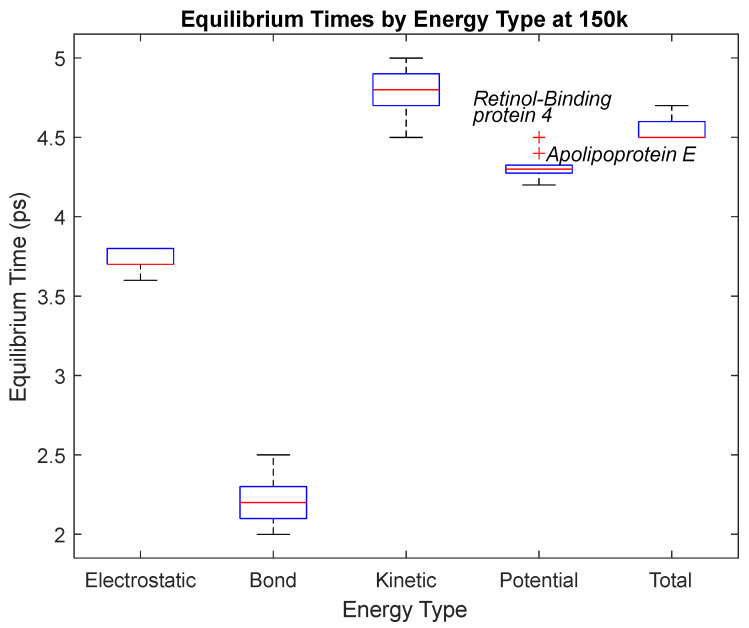
A box plot of the equilibrium times for each energy type. Data for this plot can be seen in Table 4. The red line represents the median time, the blue box is the interquartile range (25th–75th percentile), and the ends of the whiskers represent the 0th and 100th percentile, also called the maximum and minimum. Outliers are marked with red crosses and are labeled with their respective biomarkers.

**Figure 30:**
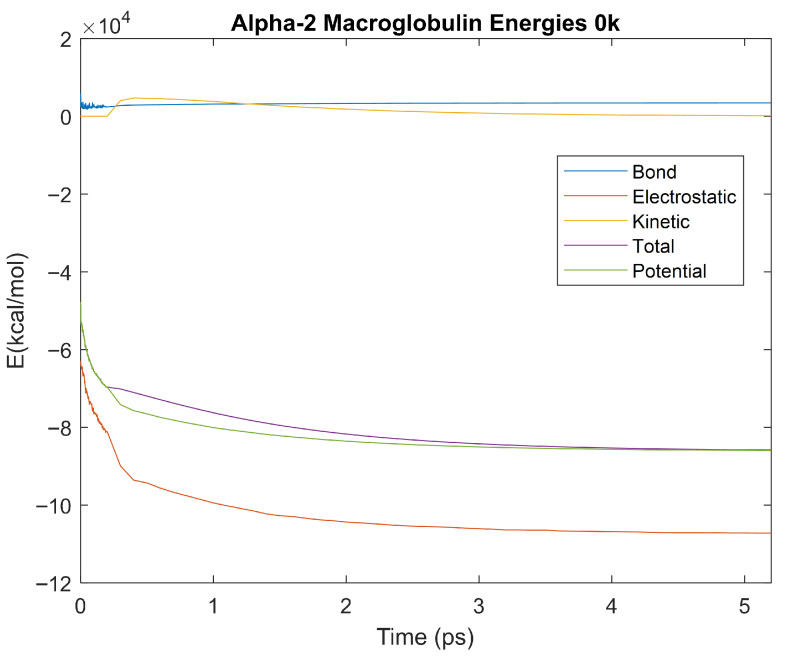
A plot of Bond, Electrostatic, Kinetic, Total, and Potential Energies for Alpha-2 Macroglobulin over the 0 K 5 ps simulation.

**Figure 31:**
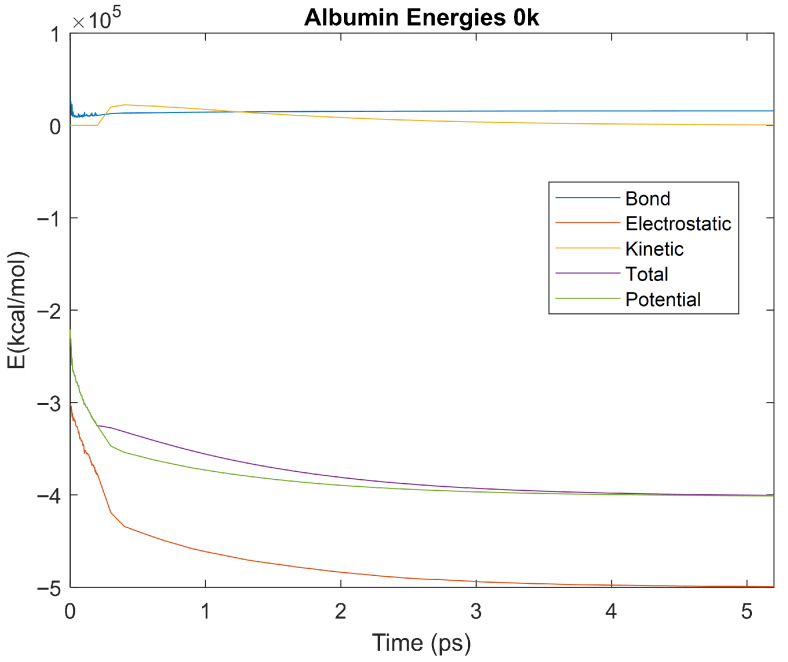
A plot of Bond, Electrostatic, Kinetic, Total, and Potential Energies for Albumin over the 0 K 5 ps simulation.

**Figure 32:**
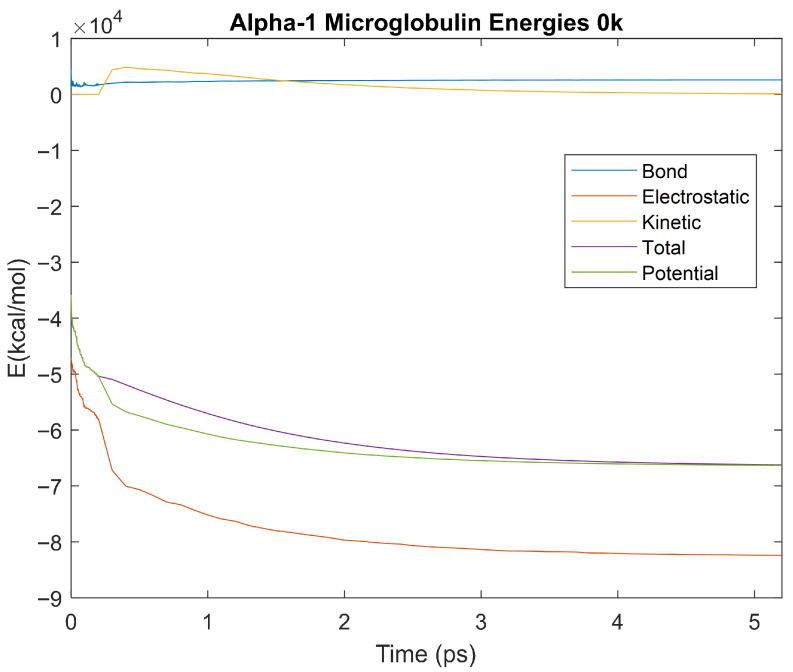
A plot of Bond, Electrostatic, Kinetic, Total, and Potential Energies for Alpha-1 Microglobulin over the 0 K 5 ps simulation.

**Figure 33:**
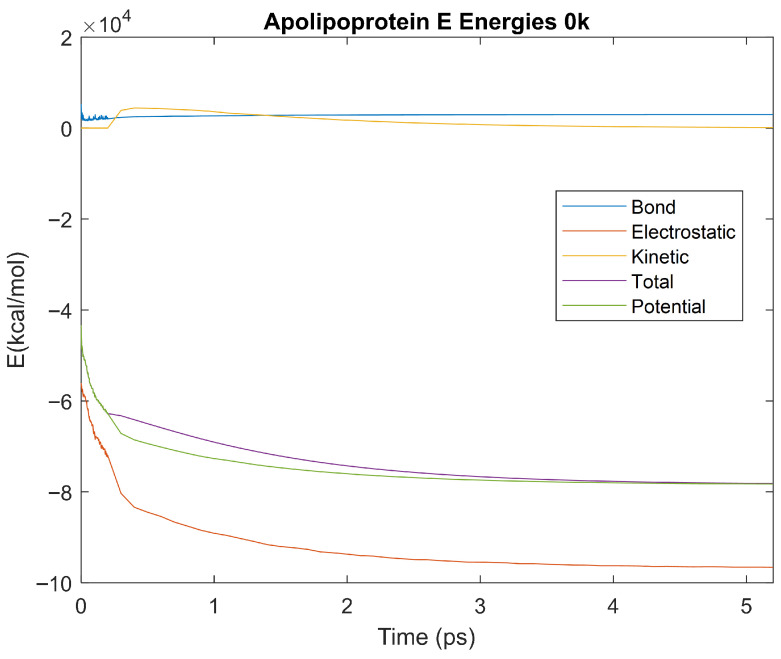
A plot of Bond, Electrostatic, Kinetic, Total, and Potential Energies for Apolipoprotein E over the 0 K 5 ps simulation.

**Figure 34:**
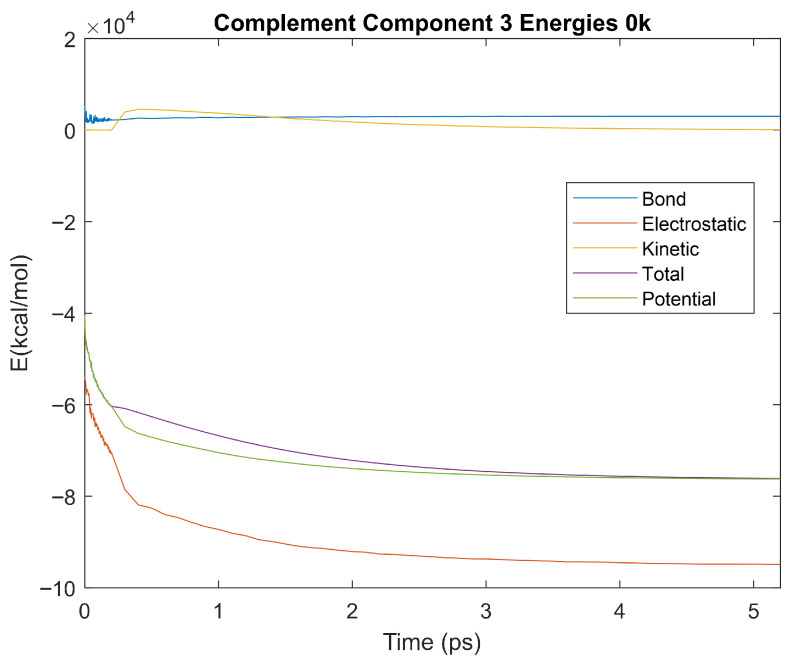
A plot of Bond, Electrostatic, Kinetic, Total, and Potential Energies for Complement Component 3 over the 0 K 5 ps simulation.

**Figure 35:**
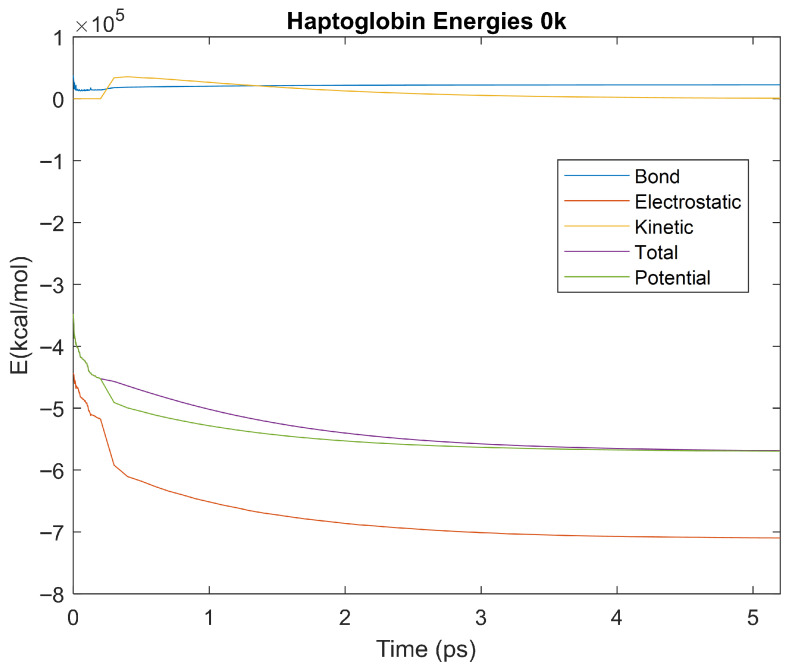
A plot of Bond, Electrostatic, Kinetic, Total, and Potential Energies for Haptoglobin over the 0 K 5 ps simulation.

**Figure 36:**
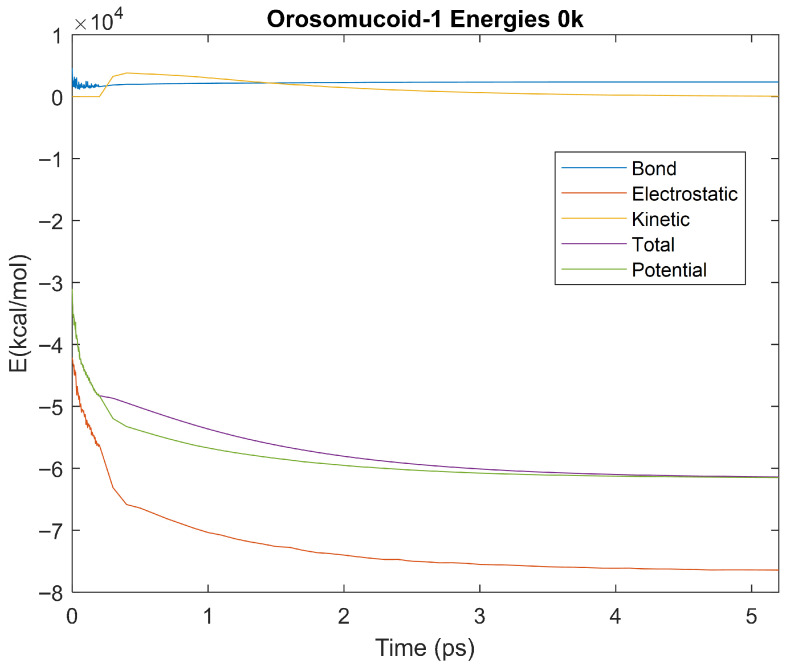
A plot of Bond, Electrostatic, Kinetic, Total, and Potential Energies for Orosomucoid-1 over the 0 K 5 ps simulation.

**Figure 37:**
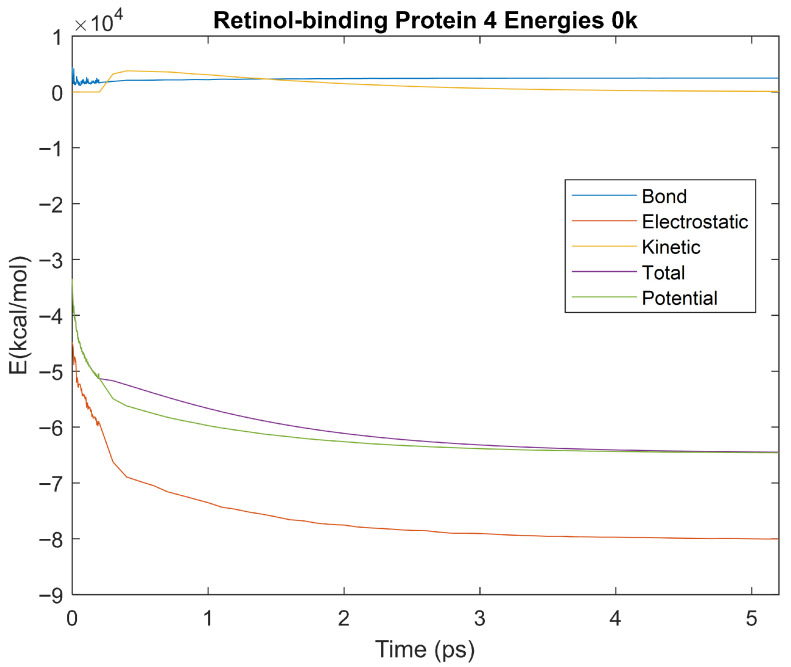
A plot of Bond, Electrostatic, Kinetic, Total, and Potential Energies for Retinol-Binding Protein 4 over the 0 K 5 ps simulation.

**Figure 38:**
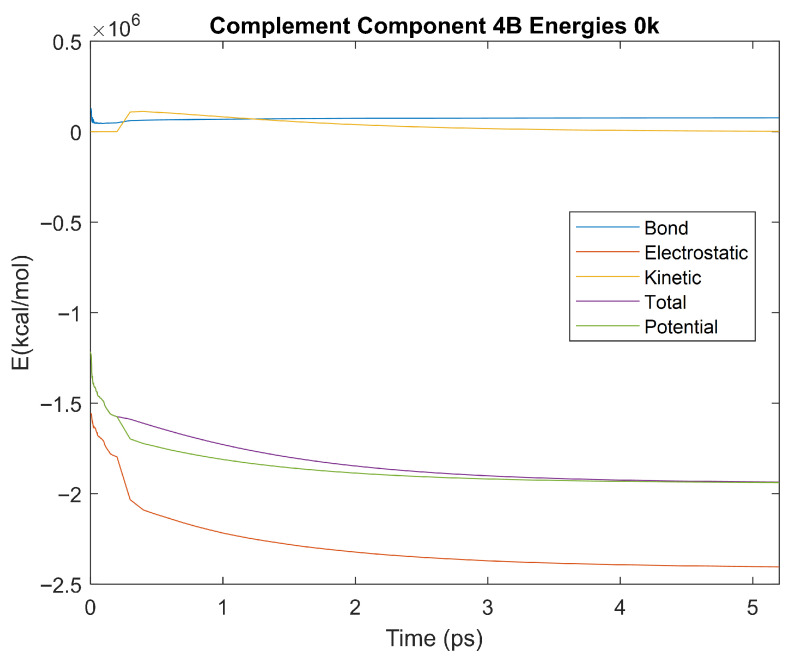
A plot of Bond, Electrostatic, Kinetic, Total, and Potential Energies for Complement Component 4B over the 0 K 5 ps simulation.

**Figure 39:**
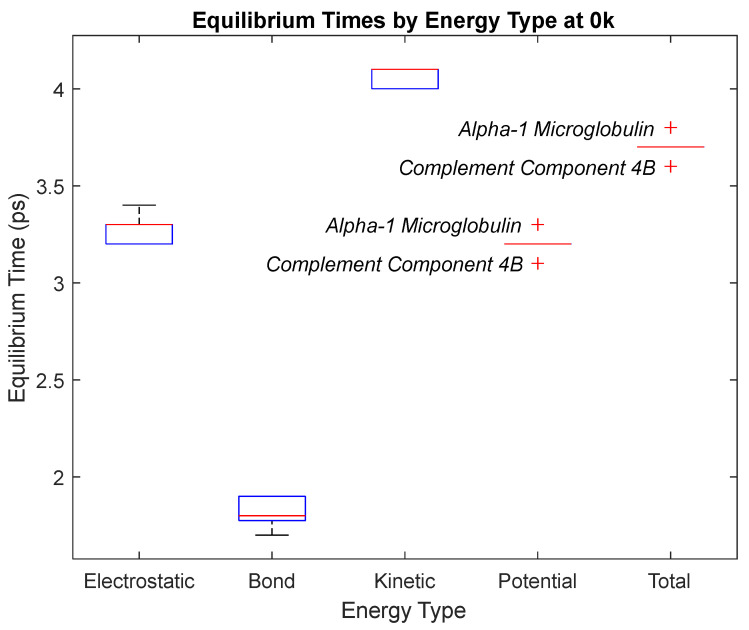
A box plot of the equilibrium times for each energy type. Data for this plot can be seen in Table 5. The red line represents the median time, the blue box is the interquartile range (25th–75th percentile), and the ends of the whiskers represent the 0th and 100th percentile, also called the maximum and minimum. Outliers are marked with red crosses and are labeled with their respective biomarker.

**Figure 40:**
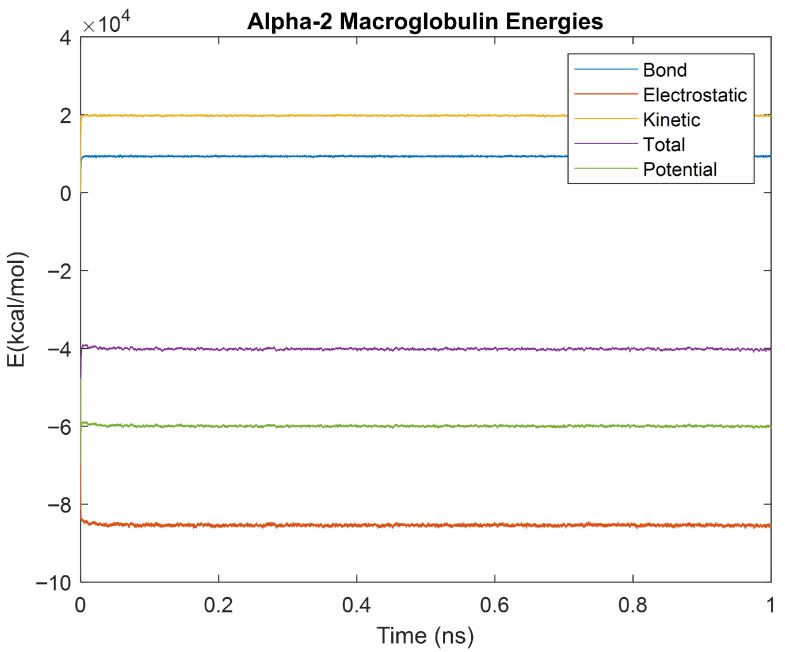
A plot of Bond, Electrostatic, Kinetic, Total, and Potential Energies for Alpha-2 Macroglobulin over the 310 K 1 ns simulation.

**Figure 41:**
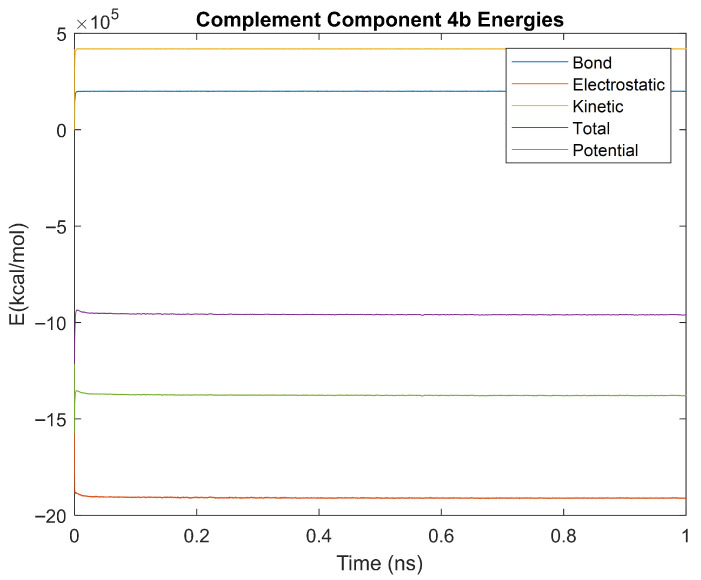
A plot of Bond, Electrostatic, Kinetic, Total, and Potential Energies for Complement Component 4B over the 310 K 1 ns simulation.

**Figure 42:**
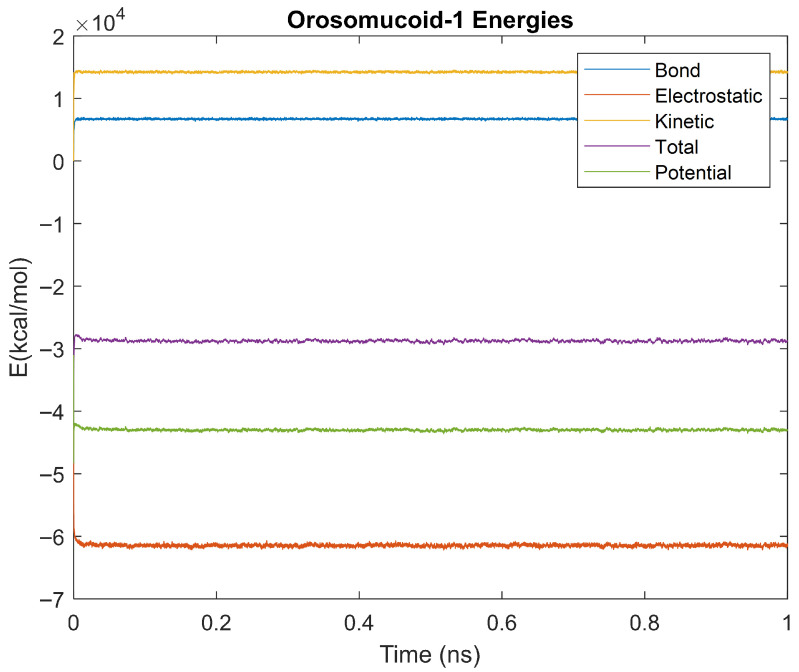
A plot of Bond, Electrostatic, Kinetic, Total, and Potential Energies for Orosomucoid-1 over the 310 K 1 ns simulation.

Simulating the proteins at lower temperatures also allows for the observation of how realistic the program’s force field parameters are. Total energy is defined as the sum of the potential energy and the kinetic energy. At 0 K, thermal motion, or kinetic energy, of a molecule ceases as kinetic energy is a linear function of temperature. Therefore, at this temperature, the kinetic energy of the molecule should be zero, and the potential energy should equal the total energy. As the temperature of the simulation increases to 150 K and 310 K, the kinetic energy should increase, and subsequently so should the total energy. Observing the plots in Figure 10, Figure 11, Figure 12, Figure 13, Figure 14, Figure 15, Figure 16, Figure 17 and Figure 18, Figure 20, Figure 21, Figure 22, Figure 23, Figure 24, Figure 25, Figure 26, Figure 27 and Figure 28, and Figure 30, Figure 31, Figure 32, Figure 33, Figure 34, Figure 35, Figure 36, Figure 37 and Figure 38, it can be seen that these fundamental thermodynamic relationships are observed. For example, Figure 36 shows the 5 ps simulation of orosomucoid-1 at 0 K. After stabilization, at the end of the 5 ps, the kinetic energy is clearly at 0 × 10^4^ kcal/mol, and both the total and potential energy are equivalent at approximately −6.1 × 10^4^ kcal/mol. Figure 26 shows the 5 ps simulation of orosomucoid-1 at 150 K. At the end of this simulation, the kinetic energy has increased to approximately 0.7 × 10^4^ kcal/mol, subsequently increasing the total energy to approximately −4.7 × 10^4^ kcal/mol and the potential energy to approximately −5.3 × 10^4^ kcal/mol for a 0.6 × 10^4^ kcal/mol difference. This highlights how the temperature increase causes the kinetic energy to increase, resulting in a total energy that is no longer equivalent to the potential energy. Lastly, Figure 16 shows the 5 ps simulation of orosomucoid-1 at 310 K. At this temperature, the kinetic energy has risen to approximately 1.3 × 10^4^ kcal/mol by the end of the simulation. Similarly, the total energy increased to approximately −3.2 × 10^4^ kcal/mol, and the potential energy rose to approximately −4.1 × 10^4^ kcal/mol for an increased 0.9 × 10^4^ kcal/mol difference. Again, the increase in temperature has increased the kinetic energy and the difference between the total and potential energy. This exemplifies the fundamental thermodynamic relationship exhibited by the force field parameters of the software that can be observed in each of the protein’s simulations.

### 5.2. One-Nanosecond Simulations

**Figure 43:**
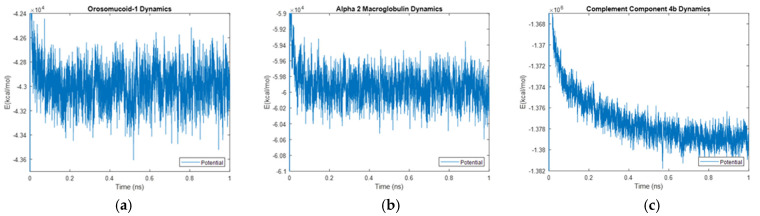
Molecular interactions in (**a**) Orosomucoid-1, (**b**) Alpha-2-Macroglobulin, and (**c**) Complement Component 4B. The dynamic potential energy drop with time in Complement Component 4B is shown in part (**c**). These studies in themselves form the basis for an accompanying article that will be submitted in the future.

Due to their differences in stability to the other biomarkers at a physiologically relevant temperature of 310 K, orosomucoid-1, alpha-2-macroglobulin, and complement component 4B were simulated for 1 ns at 310 K to gather further information. The objective of undertaking these two time-scale analyses was manyfold. A 5 ps simulation was not as computationally strenuous as a longer 1 ns simulation but was long enough to provide data on stabilization time without revealing the molecular dynamics. On the other hand, the 1 ns simulations did provide dynamics of molecular interactions as seen in Figure 43a–c where only potential energy is plotted for orosomucoid-1, alpha-2-macroglobulin, and complement component 4B, respectively. It is relevant to quantitatively determine those energies and thermodynamic stabilities with dynamics. The RMS, standard deviations, and percent changes in energy data noise from the 1 ns simulations were compared across alpha-2-macroglobulin, orosomucoid-1, and complement component 4B. Orosomucoid-1 consistently had the smallest RMS and standard deviations in its energy values compared to the other two biomarkers, while complement component 4B consistently had the largest RMS and standard deviations for all energies examined. Complement component 4B, however, had the smallest percent change (defined as the ratio of the standard deviation to the RMS value) of the three biomarkers for each energy. The percentage change for each energy was similar between alpha-2 macroglobulin and orosomucoid-1, despite their varying RMS values.

The RMS is frequently used as a metric for determining stability in molecular dynamics, as a lower RMS and standard deviation indicate less variation and point towards a protein that has a more stable equilibrium. Orosomucoid-1 consistently having a lower RMS, especially for its potential energy, supports that by this metric it is more stable than the other three simulated for 1 ns. This also appears to agree with the stabilization time assessment. However, the percent change provides a normalized way of comparing the fluctuations in the energy signal over time relative to factors such as the energy scales. This is because the size of a protein, for example, will inherently affect the energy scale of a protein, with a larger protein having a higher energy scale than a smaller protein. If a larger protein has a higher RMS than a smaller protein, it may still be more stable relative to its energy scale than the smaller protein. Thus, the percent change provides a relative metric for assessing stability or fluctuations in energy around its base signal. By this metric, complement component 4B is the most stable, followed by alpha-2-macroglobulin, then orosomucoid-1. This may explain why complement component 4B had a faster electrostatic stabilization time than the other proteins, but contradicts the prior stabilization time assessment made in regard to orosomucoid-1.

## 6. Limitations and Future Directions

This novel article provides a foundational work in the thermodynamics and molecular dynamics of biomarkers found in early OA. While this is valuable to the understanding of these biomarkers, there are limitations to the study conducted. These include duration of the simulations, replication of simulations at each temperature, subsequent statistical analysis between simulations, and wet lab validation. Regardless, granted that additional simulations and wet-lab testing are conducted, this work could inspire several clinical applications that would be valuable to OA diagnosis and prognosis.

One hypothetical clinical application relates biomarker stability to measurement viability. The faster stabilization at 310 K suggests that complement component 4B and orosomucoid-1 may be more suitable biomarkers to measure for OA, whereas the slower stabilization of alpha-2-macroglobulin suggests a less suitable biomarker for measurement. The rationale is that when biomarkers are removed from in vivo conditions, biomarkers may denature, and thus stability is needed for viable measurement. Another hypothesis relates biomarker stability to OA progression. When a molecule undergoes an oxidative reaction, the overall potential energy of that molecule changes. Therefore, a protein with a more stable potential energy would be more likely to withstand the oxidative environment of synovitis. According to Gobezie et al., the biomarkers studied in this article were found to be present in early OA [1]. As OA progresses and cartilage degrades, inflammation and oxidative stress associated with synovitis are enhanced [9,10]. Thus, biomarkers with more stable potential energies could fare better in the mid to late stages of OA than other biomarkers with less stable potential energies. For example, because the potential energy of orosomucoid-1 stabilized faster at 310 K than the other biomarkers, it might be more prevalent in the mid to later stages of OA. On the other hand, alpha-2-macroglobulin took longer to stabilize than the others and would therefore not fare well in the oxidative conditions associated with synovitis. This biomarker, however, might be more prevalent in the early onset stages of OA, as synovitis has not yet progressed. This, again, is hypothetical, as there are limitations in using information from nanosecond simulations to determine behavior in an extensive and complex condition.

A review of recent literature regarding orosomucoid-1, alpha-2-macroglobulin, and complement component 4B assists in understanding the efforts made towards the investigation of these proteins as biomarkers for OA. In one study, the proteome of synovial fluid was analyzed to observe differences between early and late-stage OA [62]. It was found that there were greater differentially expressed proteins in early-stage OA than in late-stage OA when compared to controls [62]. One of these proteins that exhibited differential expression was orosomucoid-1 [62]. Unfortunately, besides this article, the recent literature regarding orosomucoid-1 and its involvement in OA is limited. This, however, only furthers the need for research on this biomarker. A similar analysis of the osteoarthritic synovial fluid proteome found upregulation of the complement system, including complement component 4B and complement component 3 [63]. Literature suggests that this is due to their role in immune response resulting from inflammatory cytokines [63,64]. Proteomic analysis of osteoarthritic subchondral bone has identified the upregulation of alpha-2-macroglobulin, a protease inhibitor that maintains extracellular cartilage equilibrium and inhibits inflammatory processes [65,66]. Its characteristics have prompted its use as an osteoarthritic therapy via intra-articular injections [66]. Thus, these protein biomarkers and their relationship to OA prove to be of current interest to research. Recent efforts by the Osteoarthritis Biomarker Consortium have established a standardized protocol for validating potential OA biomarkers [13]. Future studies should consider these protocols when validating the clinical relevance of the biomarkers studied in this article [13].

## 7. Conclusions

The proteins studied in this paper pose as viable biomarkers for identifying the earlier stages of OA, and thus, there is a need to understand these proteins on a molecular level. By simulating these proteins in water using the molecular dynamics software NAMD and VMD, data on the thermodynamic properties of each protein were generated. This gives insight into the stability and molecular dynamics of these proteins at different temperatures and timescales. While there are some interpretive limitations, the work presented in this article is novel and provides a foundation for future work. Additionally, the research performed may inspire clinical insight, given that more advanced simulations and wet-lab validation are conducted. This could include relating stability to biomarker measurement viability and the progression of OA. Thus, this research allows for a better understanding of these biomarkers and calls for further work to advance the effort towards understanding the progression of OA.

## Figures and Tables

**Table 1:** GI number (#), specificity, sensitivity, and *p*-value for proteins found to be upregulated in OA in a complete protein analysis of synovial fluid [1].

Protein	GI #	Specificity	Sensitivity	*p* Value
Albumin	4502027	0.950	0.718	7.96 × 10^−7^
α1-Microglobulin/bikunin precursor	4502067	0.950	0.718	7.96 × 10^−7^
Fibrinogen, α chain isoform α-E preprotein	4503689	0.950	0.718	7.96 × 10^−7^
Fibrinogen, γ chain isoform γ-A precursor	4503715	1.000	0.744	1.43 × 10^−8^
α2-Macroglobulin	4557225	0.950	0.718	7.96 × 10^−7^
Apolipoprotein E	4557325	1.000	0.744	1.43 × 10^−8^
Apolipoprotein H (β2-glycoprotein I)	4557327	1.000	0.744	1.43 × 10^−8^
Complement component 3 (gel slice 3)	4557385	0.950	0.718	7.96 × 10^−7^
Complement component 3 (gel slice 5)	4557385	1.000	0.744	1.43 × 10^−8^
Ceruloplasmin (ferroxidase)	4557485	0.950	0.718	7.96 × 10^−7^
Haptoglobin	4826762	0.950	0.718	7.96 × 10^−7^
Orosomucoid 1	9257232	0.850	0.667	2.51 × 10^−4^
Group specific component (vitamin D binding protein)	32483410	1.000	0.744	1.43 × 10^−8^
Complement component 4B preprotein	50345296	1.000	0.744	1.43 × 10^−8^
PREDICTED: similar to apolipoprotein A-1 precursor	5147611	0.950	0.718	7.96 × 10^−7^
Retinol-binding protein 4, plasma precursor	55743122	0.900	0.692	1.87 × 10^−5^

Note: Adapted from [1] “High abundance synovial fluid proteome: distinct profiles in health and osteoarthritis”, by Gobezie, R., Kho, A., Krastins, B., Sarracino, D.A., Thornhill, T.S., Chase, M., Millett, P.J., Lee, D.M., 2007, *Arthritis research & therapy*, *9*(2): R36.

**Table 2:** Properties of the protein biomarkers that were simulated using the VMD and NAMD software. The gene and weight for each protein were gathered from the UniProt data bank [35,38,39,40,42,44,46,48,50]. Amino acid length was gathered via the GI number from the NCBI databank [51,52,53,54,55,56,57,58,59].

Protein	Gene	Weight (Da)	Amino Acid Length (From GI #)	Citation
Albumin	ALB	69,367	609	[35,51]
α1-Microglobulin	AMBP (HCP, ITIL)	38,999	352	[38,52]
α2-Macroglobulin	A2M (CPAMD5)	163,291	1474	[39,53]
Apolipoprotein E	APOE	36,154	317	[40,54]
Complement component 3	C3 (CPAMD1)	187,148	1663	[42,55]
Haptoglobin	HP	45,205	406	[44,56]
Orosomucoid 1	ORM1 (AGP1)	23,540	201	[46,57]
Complement component 4B preprotein	C4B (CO4, CPAMD3)	192,751	1744	[48,58]
Retinol-binding protein 4, plasma precursor	RBP4	23,010	201	[50,59]

**Table 3:** The time taken in picoseconds for each of the protein biomarkers to reach equilibrium for each respective energy at 310 K.

Amount of Time (ps) Taken to Reach Equilibrium (310 K)					
Biomarker	Electrostatic	Bond	Kinetic	Potential	Total
Albumin	2.4	3.1	2.7	2.2	2.3
α1-Microglobulin	4.1	2.9	2.5	2.4	2.4
α2-Macroglobulin	2.7	3.9	3.2	3.5	3.1
Apolipoprotein E	3.6	4.1	3.5	2.3	2.4
Complement component 3	3.1	3.5	2.6	2.1	2.2
Haptoglobin	3.2	3.7	2.5	2.3	2.3
Orosomucoid 1	2.9	3.9	3.3	1.7	2.0
Complement component 4B	0.8	3.2	2.9	2.7	2.7
Retinol-binding protein 4	3.3	3.9	2.7	2.3	2.2

**Table 4:** The time taken in picoseconds for each of the protein biomarkers to reach equilibrium for each respective energy at 150 K.

Amount of Time (ps) Taken to Reach Equilibrium (150 K)					
Biomarker	Electrostatic	Bond	Kinetic	Potential	Total
Albumin	3.6	2.2	4.8	4.2	4.5
α1-Microglobulin	3.8	2.3	4.7	4.3	4.5
α2-Macroglobulin	3.8	2.1	4.7	4.3	4.5
Apolipoprotein E	3.7	2.1	4.9	4.4	4.6
Complement component 3	3.8	2.5	4.5	4.3	4.5
Haptoglobin	3.7	2.1	4.8	4.3	4.6
Orosomucoid 1	3.8	2.3	4.9	4.3	4.6
Complement component 4B	3.7	2.2	4.8	4.2	4.5
Retinol-binding protein 4	3.7	2	5	4.5	4.7

**Table 5:** The time taken in picoseconds for each of the protein biomarkers to reach equilibrium for each respective energy at 0 K.

Amount of Time (ps) Taken to Reach Equilibrium (0 K)					
Biomarker	Electrostatic	Bond	Kinetic	Potential	Total
Albumin	3.2	1.8	4.1	3.2	3.7
α1-Microglobulin	3.3	1.9	4.0	3.3	3.8
α2-Macroglobulin	3.2	1.7	4.1	3.2	3.7
Apolipoprotein E	3.2	1.8	4.1	3.2	3.7
Complement component 3	3.3	1.7	4.1	3.2	3.7
Haptoglobin	3.3	1.9	4.0	3.2	3.7
Orosomucoid 1	3.3	1.8	4.1	3.2	3.7
Complement component 4B	3.4	1.9	4.0	3.1	3.6
Retinol-binding protein 4	3.2	1.9	4.1	3.2	3.7

**Table 6:** Values obtained from the analysis of the energy data noise for alpha-2-macroglobulin. Energy data between 0.1 ns and 1 ns from the 1 ns simulation was considered in the analysis.

Alpha-2 Macroglobulin Energy Noise (0.1–1 ns)					
	Bond	Electrostatic	Kinetic	Total	Potential
RMS (kcal/mol):	9320.9968	85,387.3186	19,778.495	40,165.0394	59,942.976
Standard Deviation (kcal/mol):	102.8123	231.4206	110.415	193.1473	160.236
Percent Change (kcal/mol):	0.01103	0.0027102	0.0055826	0.0048088	0.0026731

**Table 7:** Values obtained from the analysis of the energy data noise for complement component 4B. Energy data between 0.1 ns and 1 ns from the 1 ns simulation was considered in the analysis.

Complement Component 4B Energy Noise (0.1–1 ns)					
	Bond	Electrostatic	Kinetic	Total	Potential
RMS (kcal/mol):	199,128.5033	1,909,673.714	418,972.4289	958,798.3667	1,377,769.945
Standard Deviation (kcal/mol):	478.31444	1578.9485	507.70323	1636.9161	1533.8466
Percent Change (kcal/mol):	0.002402	0.00082682	0.0012118	0.0017073	0.0011133

**Table 8:** Values obtained from the analysis of the energy data noise for orosomucoid-1. Energy data between 0.1 ns and 1 ns from the 1 ns simulation was considered in the analysis.

Orosomucoid-1 Energy Noise (0.1–1 ns)					
	Bond	Electrostatic	Kinetic	Total	Potential
RMS (kcal/mol):	6711.66627	61,459.1381	14,222.678	28,782.0271	43,004.1156
Standard Deviation (kcal/mol):	87.60533	198.0308	95.3199	168.9017	139.2274
Percent Change (kcal/mol):	0.013053	0.0032222	0.006702	0.0058683	0.0032375

## Data Availability

The original contributions presented in this study are included in the article. Further inquiries can be directed to the corresponding author.
